# The core bacteriobiome of Côte d’Ivoire soils across three vegetation zones

**DOI:** 10.3389/fmicb.2023.1220655

**Published:** 2023-08-24

**Authors:** Chiguié Estelle Raïssa Amon, Romain Kouakou Fossou, Anicet E. T. Ebou, Dominiqueua K. Koua, Claude Ghislaine Kouadjo, Yao Casimir Brou, Don Rodrigue Rosin Voko Bi, Don A. Cowan, Adolphe Zézé

**Affiliations:** ^1^Laboratoire de Biotechnologies Végétale et Microbienne, UMRI Sciences Agronomiques et Génie rural, Institut National Polytechnique Félix Houphouët-Boigny, Yamoussoukro, Côte d'Ivoire; ^2^Laboratoire Central de Biotechnologies, Centre National de la Recherche Agronomique, Abidjan, Côte d’Ivoire; ^3^Unité de Formation et de Recherche en Agroforesterie, Université Jean Lorougnon Guédé, Daloa, Côte d’Ivoire; ^4^Centre for Microbial Ecology and Genomics, Department of Biochemistry, Genetics and Microbiology, University of Pretoria, Pretoria, South Africa

**Keywords:** microbiome, common core bacteriobiome, bacteria, 16S rDNA, Côte d’Ivoire

## Abstract

The growing understanding that soil bacteria play a critical role in ecosystem servicing has led to a number of large-scale biogeographical surveys of soil microbial diversity. However, most of such studies have focused on northern hemisphere regions and little is known of either the detailed structure or function of soil microbiomes of sub-Saharan African countries. In this paper, we report the use of high-throughput amplicon sequencing analyses to investigate the biogeography of soil bacteria in soils of Côte d’Ivoire. 45 surface soil samples were collected from Côte d’Ivoire, representing all major biomes, and bacterial community composition was assessed by targeting the V4-V5 hypervariable region of the 16S ribosomal RNA gene. Causative relationships of both soil physicochemical properties and climatic data on bacterial community structure were infered. 48 phyla, 92 classes, 152 orders, 356 families, and 1,234 genera of bacteria were identified. The core bacteriobiome consisted of 10 genera ranked in the following order of total abundance: *Gp6*, *Gaiella*, *Spartobacteria_genera_incertae_sedis*, *WPS-1_genera_incertae_sedis*, *Gp4*, *Rhodoplanes*, *Pseudorhodoplanes*, *Bradyrhizobium*, *Subdivision3_genera_incertae_sedis*, and *Gp3*. Some of these genera, including *Gp4* and *WPS-1_genera_incertae_sedis*, were unequally distributed between forest and savannah areas while other taxa (*Bradyrhizobium* and *Rhodoplanes)* were consistently found in all biomes. The distribution of the core genera, together with the 10 major phyla, was influenced by several environmental factors, including latitude, pH, Al and K. The main pattern of distribution that was observed for the core bacteriobiome was the vegetation-independent distribution scheme. In terms of predicted functions, all core bacterial taxa were involved in assimilatory sulfate reduction, while atmospheric dinitrogen (N_2_) reduction was only associated with the genus *Bradyrhizobium*. This work, which is one of the first such study to be undertaken at this scale in Côte d’Ivoire, provides insights into the distribution of bacterial taxa in Côte d’Ivoire soils, and the findings may serve as biological indicator for land management in Côte d’Ivoire.

## 1. Introduction

Soils harbour complex microbial communities (Schloss and Handelsman, 2005; Pivato et al., 2015) with the capacity for diverse biogeochemical functions (Lavelle and Spain, 2001; Fenchel et al., 2012). Soil microbiomes provide an interface between soil nutrients and plant nutrient-uptaked mechanisms, and therefore represent key factors in soil health (Sindhu et al., 2019), nutrient cycling and plant health (Fierer, 2017; Jacoby et al., 2017). Soil microbiomes may therefore act as regulators of plant productivity, especially in nutrient-poor ecosystems where plant symbionts are responsible for the uptake of limiting nutrients (Quoreshi, 2008; Sindhu et al., 2019).

A number of studies have investigated microbial community compositions, and the impact of environmental factors on microbiome composition, on a national, continental or global scale (Fierer and Jackson, 2006; Delgado-Baquerizo et al., 2018; Karimi et al., 2018; Gnangui et al., 2021; Cowan et al., 2022). It has been shown that different environmental factors lead to different niches and the specific selection of a core microbiome (Turnbaugh et al., 2007; Liu et al., 2018; Pajares et al., 2018; Koutika et al., 2020). Its influence on soil health, plant growth and agricultural sustainability has been assessed in different ecosystems (Zhao et al., 2019; Castellano-Hinojosa and Strauss, 2021).

Several studies using high-throughput sequencing facilities to assess the functions and drivers of the core microbiome in soils have been conducted at small scale and at local levels (Pershina et al., 2018; Jiao et al., 2022; Kolton et al., 2022). Recent studies of African soil microbiomics have attempted to map both the microbial diversity and functional capacity of soil microbial communities at a local (Maquia et al., 2020; Gnangui et al., 2021) or continental scales (Cowan et al., 2022). However, the authors are unaware of any biogeographical studies focusing on the causative relationships that may exist between environmental factors and the core soil microbiome structure in any sub-Saharan nation.

Côte d’Ivoire can be divided into three main agro-ecological biomes (Ducroquet et al., 2017). The Guinean biome is located in the southern part of the country and represents more than 48% of the national territory (Ducroquet et al., 2017). This is the highest rainfall agro-ecological region and includes the forest zone (FAO, 2009). The climate has a equatorial/subequatorial type with a long rainy season that supports evergreen forest and semi-deciduous humid forest types (Guillaumet and Adjanohoun, 1971; Kassin et al., 2017). The main crop types grown in this area are cash (cocoa, coffee) and food crops (cassava, rice, plantain) (Ducroquet et al., 2017). Here, the pH was often more acidic in the top surface, with a sandy-loam texture (Tondoh et al., 2015; Kassin et al., 2017). At higher latitudes, the soils contain variable and often significant proportions of basic cations (Ca^2+^, Mg^2+^, K^+^, and Na^+^), as well as high amounts of nitrogen, leading to an increase in the rate of organic matters decomposition (Avenard et al., 1971).

The Forest-Savannah transition zone, which corresponds to the Sudano-Guinean biome, is centrally located and represents 19% of the nation’s land mass (Ducroquet et al., 2017).This region is characterized by a humid tropical climate within which a transition takes place between the southern forest zone and the north, dominated by the savannah is the transition zone is characterized by a relatively low rainfall and a mixed landscape where the two types of vegetation coexist, termed the forest-savannah mosaic (Gautier, 1990).

The Sudanian biome, located in the north, occupies about 33% of the country’s land mass (Ducroquet et al., 2017). This biome is characterized by both a subhumid and semi-arid tropical climate with a longer dry season (Guillaumet and Adjanohoun, 1971; Tano et al., 2020). Nitrogen content have been found to be relatively low (Assémien et al., 2017) with soil pH values varying between 5.7 and 6.4 (Tano et al., 2020).

Despite recent studies on microbiome composition in Côte d’Ivoire soils (Assémien et al., 2017; Kouadio et al., 2017; Séry et al., 2018; Gnangui et al., 2021), nothing is known of the core microbiome distribution across the nation, nor of the putative functions and ecosystem services of these microbiome. The main objectives of this work were therefore: (i) to examine the distribution of bacterial taxa across Côte d’Ivoire soils by high-throughput 16S ribosomal RNA (rRNA) gene sequencing; (ii) to identify the principal environmental factors that may influence the distribution of bacterial communities, at the phylum and genus levels, and (iii) to predict the potential metabolic profiles of the core bacteriobiome across Côte d’Ivoire soils.

## 2. Methods

### 2.1. Experimental design

**Figure 1:**
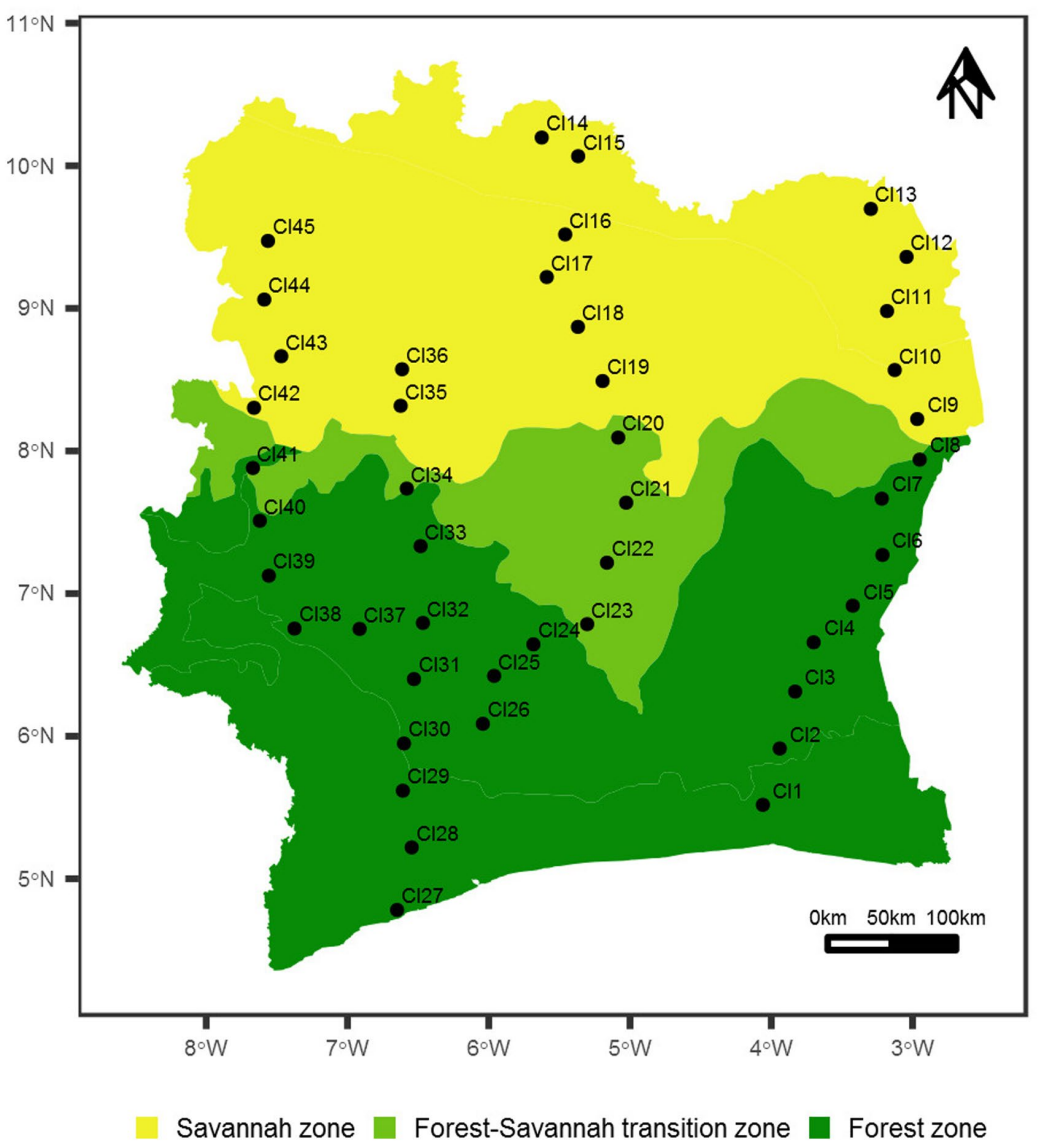
Map of Côte d’Ivoire showing the 45 sampled sites. Geographic coordinates of the sampled sites are given in Supplementary Table S1. The map is colored according to the main vegetation types in Côte d’Ivoire (forest, savannah and forest-savannah transition zones).

Forty-five (45) surface soil samples were recovered across all the major biomes in Côte d’Ivoire from August to September 2017 (Gnangui et al., 2021; Cowan et al., 2022; Figure 1). Twenty-three (23) soil samples were collected in the forest zone, which represent more than 48% of the national territory, while 17 and 5 samples were collected in the savannah and the forest-savanna transition zones, respectively. Each sampling point was represented as a virtual rectangle of 100 × 50 meters, at the corners of which soil subsamples (0–5 cm depth: approx. 25 g each) were recovered into sterile plastic Whirlpak® bags. Geographical coordinates as well as climatic and environmental data of the study locations were recorded (Supplementary Table S1). Soils were sub-divided for chemical analysis (stored at −4°C) and eDNA extraction (stored at −80°C).

### 2.2. Soil physico-chemical analyses

Soil physico-chemical characteristics of the soil samples were determined by Bemlab (Strand, Cape Province, South Africa) using standard methods (Gnangui et al., 2021; Cowan et al., 2022). Prior to analyses, the samples were air-dried at room temperature for 4 days, separated from roots and debris, and passed through a 2 mm sieve. The sieved replicate samples of each sampling site were pooled to obtain a composite soil sample. All analyses were performed using the composite soil samples as described elsewhere (Gnangui et al., 2021; Cowan et al., 2022).

### 2.3. Total DNA extraction, PCR and high throughput sequencing

Total DNA extraction, amplification and Illumina MiSeq amplicon sequencing were carried out as previously reported (Cowan et al., 2022). After DNA extraction at the Centre for Microbial Ecology and Genomics (University of Pretoria, South Africa), DNA sequencing was done at the MRDNA sequencing facility (www.mrdnalab.com, TX, United States). The V4–V5 variable region of the 16S rRNA gene was amplified and sequenced using primers: 515F-Y (5′-GTGYCAGCMGCCGCGGTAA-3′; Parada et al., 2016) and 909–928R (5′-CCCCGYCAATTCMTTTRAGT-3′; Wang and Qian, 2009), with 12 nucleotides unique barcode at 5-end of 515F-Y for each soil sample.

### 2.4. Bioinformatics and statistical analysis for microbial community analyses

The sequences obtained from the Illumina MiSeq platform were analyzed using Qiime 2 v2018.6 (Bolyen et al., 2019). Sequences were initially demultiplexed according to sample barcodes. Sequence processing was done as in Cowan et al., with some modifications (Cowan et al., 2022). A pre-processing step was performed to remove samples barcodes, followed by a filtering performed using the cutadapt v2.10 function under Linux (Martin, 2011) to delete forward primers as well as incomplete sequences. The resulting fastq file was then separated into several files based on the sequences size. A second filtering step was then done on each fastq file via DADA2 algorithm under Qiime 2, allowing the removal of low quality sequences, as well as reverse primers (Callahan et al., 2016; Christensen et al., 2018). The denoised sequences were clustered into Amplicon Sequences Variant (ASVs) with DADA2 (Callahan et al., 2016). The resulting ASVs were classified using the naive Bayesian Ribosomal Database Project (Wang et al., 2007; Cole et al., 2014) with the latest RDP classifier release 2.13 (July 2020^1^). The resulting taxonomic table files were then merged into one file for further analyses. A last filtering has been done on this file to remove 16S sequences affiliated to Archaea and chloroplasts.

All statistical analyses were performed in R v4.0.3 (R Core Team, 2020) with raster v3.5–15 (Hijmans, 2020), vegan v2.5–7 (Oksanen et al., 2020), phyloseq v1.34.0 (McMurdie and Holmes, 2013) and ggplot2 v3.3.6 (Wickham, 2016). Sequences were deposited in the Sequence Read Archive (SRA) of the National Center for Biotechnology Information (NCBI) database under BioProject PRJNA695288.

Soils texture classes were determined according to the standard USDA particle-size classification as in the soil texture R package (Moeys, 2018). Rarefaction curves were produced using the rarecurve function from the vegan package. The Kruskal-Wallis test was used to assess the statistical differences among the three type of vegetation for each of the variables considered in the study, followed by a Wilcoxon rank sum test (with a Benjamini-Hochberg correction) when there were significant differences. Based on their relative abundances, phyla were classified according to a bottom-up hierarchical clustering using Ward’s method (Murtagh and Legendre, 2014). Comparisons following Wilcoxon rank sum test among phyla identified those that were abundant (major taxa) with more than 1% of relative abundance or least represented (minor and rare taxa, less than 1% of relative abundance) (Karimi et al., 2018). Raster package was used to map major phyla across Côte d’Ivoire soils, based on phylum abundance. The distribution of the dominant bacterial taxa across the three biomes was determined using the analysis of similarity (ANOSIM) and a variance partitioning performed on physico-chemical, climatic, spatial and land use variables as described elsewhere (Karimi et al., 2018). To assess the contribution of physico-chemical, climatic and spatial variables in the distribution of each core phylum in soils, a redundancy analysis (RDA) was performed as done elsewhere (Shen et al., 2018; Alami et al., 2020). On the total of 16 variables considered, four (i.e., Ca, Mg, N and sand) were removed to avoid collinearity. For each phylum, the most relevant variables were selected by the RDA, with an indication about their adjusted *R*^2^ and *p*-values. The statistical significance of the results obtained was tested by an analysis of variance (ANOVA) with 999 permutations. Coefficient correlations between each core phylum and significant variables chosen by the RDA were obtained through Hmsic package (Harrell Jr, 2021). The resulting dataset, including adjusted *R*^2^, *p*-values and correlation coefficients for each core phylum was used to build a bubbleplot with ggplot. The core bacteriobiome of all 45 soils samples was identified at the genus level based on a relative abundance of >0.5 and 95% occurrence in soil samples (Neu et al., 2021). Differences in the distribution of the core bacteriobiome according to the soil samples and the three types of vegetation were evaluated by performing a Kruskal-Wallis test, followed by a Wilcoxon rank sum test with a significant level of *p* < 0.05. RDA analysis was carried out to show variables involved in the distribution of the core genera. Spearman correlations among the abundance of each core genus and environmental variables were done using the Hmsic package (Harrell Jr, 2021). Only correlations greater than 0.5 or less −0.5 with a *p* < 0.01 were considered. A correlogram for correlation levels among the core genera were built by calculating Pearson correlation.

### 2.5. Core microbiome function prediction using PICRUSt2

The filtered ASV matrix was used to predict the core bacterial metabolic functions from PICRUSt2 v2.4.2 (Douglas et al., 2020; Djemiel et al., 2022) with implemented tools HMMER^2^ (Finn et al., 2011), SEPP (SATe-enabled Phylogenetic Placement) (Mirarab et al., 2012), GAPPA (Genesis Applications for Phylogenetic Placement Analysis) (Czech et al., 2020) and Castor (Louca and Doebeli, 2018). The KEGG Orthology (KO) was used^3^ (Kanehisa and Goto, 2000; Kanehisa, 2019; Kanehisa et al., 2021, 2022; Djemiel et al., 2022) and KO numbers obtained from PICRUSt2 analysis was used to map the different functions categories. A manual selection was made among all the metabolic pathways predicted by ASV and only those of interest in agriculture were selected, i.e., nitrogen metabolism, sulfur metabolism, carbon fixation as well as symbiotic pathway (Yan et al., 2015). Heatmaps of the metabolic pathways were generated with the ggplot function. Kruskal-Wallis tests were carried out to determine differences between functions predicted across the samples and the types of vegetation.

## 3. Results

### 3.1. Physico-chemical and climate characteristics of Côte d’Ivoire soils

pH values of the 45 soils ranged from 4.9 to 7.8, being consistent with pH range expected in soil of tropical and humid regions, as already reported for Côte d’Ivoire soils (Tondoh et al., 2015; Kassin et al., 2017; Tano et al., 2020). Approximately 11% of soils were classified as strongly acidic (pH <5.5), 64% as acidic (5.5 to 6.5), 22% neutral (6.5 to 7.5) and only 2% as alkaline (pH over 7.5). Acidic soils were observed across the three vegetation biomes, together with the neutral soils. In contrast, the strongly acidic soils were mostly encountered in the forest zone. The majority of these soils (at least 90%) had a high sand content with a minimum of 53%. Of all the soils, 47% were sandy loam, 13% loam sandy, 31% sandy clay loam and 9% loam.

Chemical analyses revealed that organic carbon and total nitrogen contents of soils were statistically different among the three types of vegetation (*p* < 0.01), with a high level in forest soils (1.4 ± 0.46 for C and 0.16 ± 0.06 for N), while savannah and the contact zone had similar C and N contents. pH values of soils, and all the chemical elements tested were not different between the three vegetation zones. Similar results were observed for the proportion of sand, silt and clay. Precipitation (*p* = 3.9e-03) and temperature (*p* = 2e-03) data were statistically different among the three vegetation zones. All of these data are presented in Supplementary Tables S1, S2.

### 3.2. Bacterial communities in Côte d’Ivoire soils are mainly dominated by three ecologically important phyla

**Figure 2:**
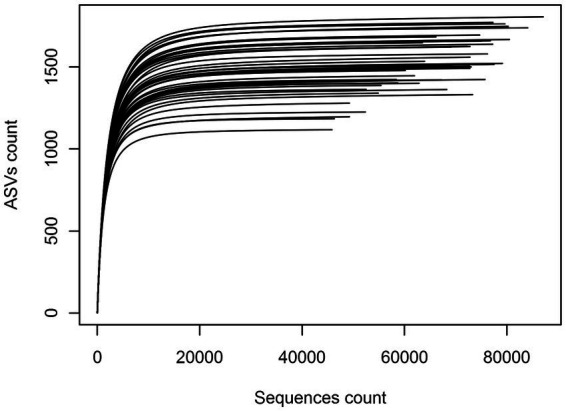
Rarefaction curve of the forty-five samples showing the number of ASVs recorded as a function of sequences count.

**Figure 3:**
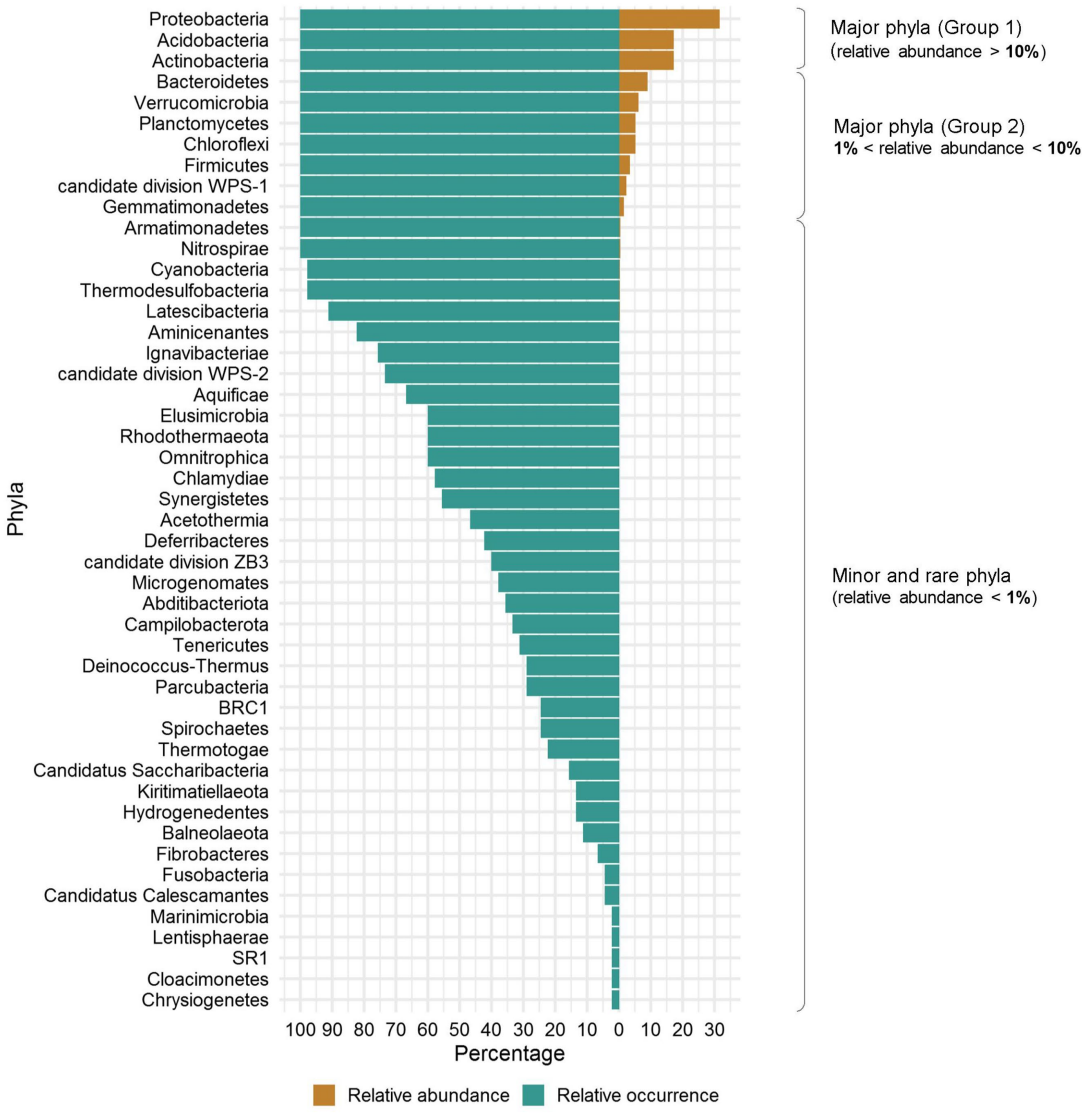
Classification of bacterial phyla from Côte d’Ivoire soils according to their abundance and relative occurrence. The occurrence varies between 100% for a total occupation of the soils and about 2% for a presence in a single soil.

To assess sampling quality, rarefaction curves were plotted, showing all the curves reaching asymptotes with less than 30,000 sequences and suggesting that the sequencing effort of each eDNA sample was sufficient (Figure 2). Of the total of 3,645,012 sequences obtained, the 3,175,778 remaining after filtering were clustered and yielded a total of 23,910 bacterial ASVs. Variation in sequences length was shown in Supplementary Table S3. Subsequent analyses allowed their clustering into 48 Phyla, 92 Classes, 152 Orders, 356 Families and 1,234 Genera. When a hierarchical classification of the 48 phyla was performed according to their abundance (Supplementary Figure S1), it was shown that the cumulative sequences of 10 phyla represented more than 98% of the total filtered sequences. Their abundance and occurrence in soils were significantly different (*p* < 0.05) from those of the remaining 37 phyla (Supplementary Table S4). These 10 core phyla included Acidobacteria, Actinobacteria, Bacteroidetes, Chloroflexi, Firmicutes, candidate division WPS-1, Gemmatimonadetes Planctomycetes, Proteobacteria, and Verrucomicrobia. According to their relative abundance, they could be divided into two main groups: a first group of phyla with a relative abundance per phylum above 10% (i.e., Acidobacteria, Actinobacteria, and Proteobacteria) and a second group of phyla with a relative abundance per phylum ranging from 1 to 10% (the seven remaining) (Figure 3). The Proteobacteria, Actinobacteria, Bacteroidetes and Chloroflexi communities were differently distributed across the three biomes (ANOSIM: 0.25 < *R* < 0.5, *p* < 0.05) while the other phyla (e.g., Acidobacteria, Verrucomicrobia etc.) were equally distributed in the three biomes (ANOSIM: 0.1 < *R* < 0.25, *p* < 0.05). The Proteobacteria and Bacteroidetes for example were more spread in the forest zone of Côte d’Ivoire, while Chloroflexi were more abundant in the savannah zone. Supplementary Figure S2 shows the maps of the 10 dominant phyla based on their relative abundance and occurrence in Côte d’Ivoire soils.

### 3.3. Causative relationships between environmental factors and bacterial phyla distribution in Côte d’Ivoire soils

**Figure 4:**
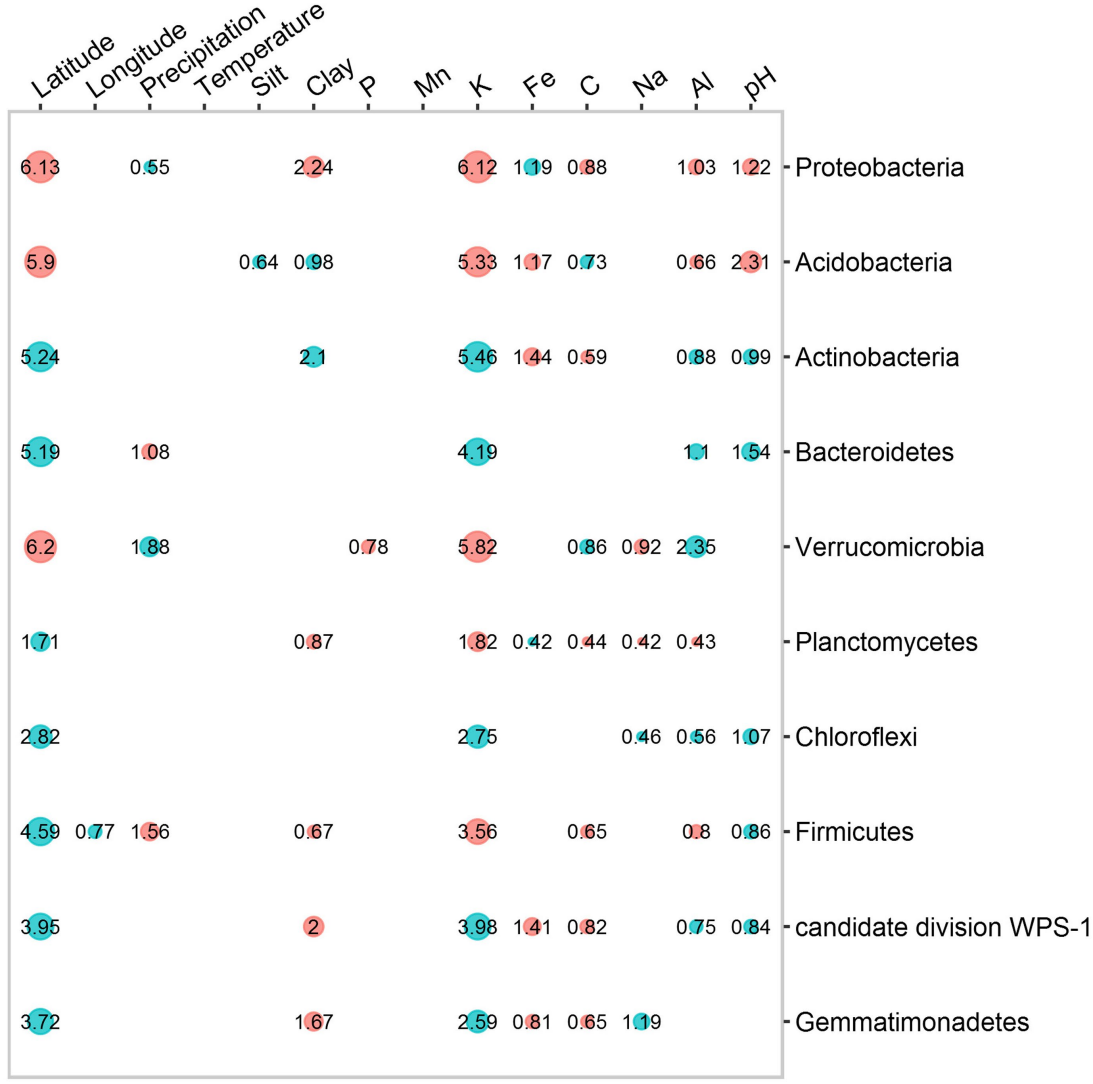
Contribution and effects of each variable (geographical location, climate and physico-chemical properties) in the distribution of bacterial taxa. The proportions of variance explained were the significant contributions of these variables (*p* < 0.05). The size of the circles as well as the values written inside provide information on the proportion of variance explained by each variable. The colors blue and red indicate the effect of the regression coefficients based on Spearman’s correlation (blue, positive effect, red, negative effect).

Redundancy analysis was carried out on soil physico-chemical properties, as well as climate and spatial variables, in order to understand their influence on bacterial community distribution. The total variance explained by all these variables in the distribution of the 10 phyla ranged from 6.12 to 19.36%. Their effect on bacterial phylum distribution is summarized by the following relationships: physicochemical properties > spatial data > climate (Figure 4). Among soil nutrients, potassium (K) content had a significant impact on the 10 phyla. For example, the abundance of Proteobacteria (*R*^2^ = 6.1%), Verrucomicrobia (*R*^2^ = 5.8%) and Acidobacteria (*R*^2^ = 5.3%) decreased with increasing K levels, but the opposite effect was observed on Actinobacteria (*R*^2^ = 5.5%) and Bacteroidetes (*R*^2^ = 6.2%). Together with K content, soil pH was one of the factors that significantly influenced all the 10 phyla (*p* < 0.05). Al levels had a higher effect on Verrucomicrobia (*R*^2^ = 2.3%) than on the other phyla. Concerning the spatial variables, only latitude had a significant effect on all phyla distribution, with a higher influence on Verrucomicrobia (*R*^2^ = 6.2%), Proteobacteria (*R*^2^ = 6.1%) and Acidobacteria (*R*^2^ = 5.9%) (Figure 4).

### 3.4. The core bacteriobiome of Côte d’Ivoire soils

The core bacteriobiome was determined at the genus level. Among the total of 1,234 genera, only 10 were present with 100% occurrence (Supplementary Table S5) and above 0.5% relative abundance in all samples (Supplementary Table S5; Supplementary Figure S3). The 10 core genera belonged to the phyla of Proteobacteria (30%), Acidobacteria (30%), Verrucomicrobia (20%), Actinobacteria (10%) and candidate division WPS-1 (10%) (Table 1).

**Table 1:** Supplementary Table summarizing of occurrence and abundance data of the core genera found in Côte d’Ivoire soils.

Phyla	Genera	Occurrence (%)	Min.-Max. soil relative abundance (%)	Median (%)
Actinobacteria	*Gaiella*	100	1.1–10.9	3.6
Acidobacteria	*Gp3*	100	0.5–3.1	1.1
	*Gp4*	100	0.5–5.4	2.0
	*Gp6*	100	1.8–12.8	5.4
Proteobacteria	*Pseudorhodoplanes*	100	1.0–5.6	1.8
	*Rhodoplanes*	100	1.3–4.5	2.1
	*Bradyrhizobium*	100	0.5–4.0	1.9
Verrucomicrobia	*Spartobacteria_genera_incertae_sedis*	100	0.7–10.7	3.5
	*Subdivision3_genera_incertae_sedis*	100	0.6–5.5	1.5
candidate division WPS-1	*WPS-1_genera_incertae_sedis*	100	0.8–4.6	2.2

Each genus was related to its phylum in the first column. Relative occurrence values are given for each genus. In addition to median values, minimum and maximum relative abundance from all soils are given to show the range of variation.

**Figure 5:**
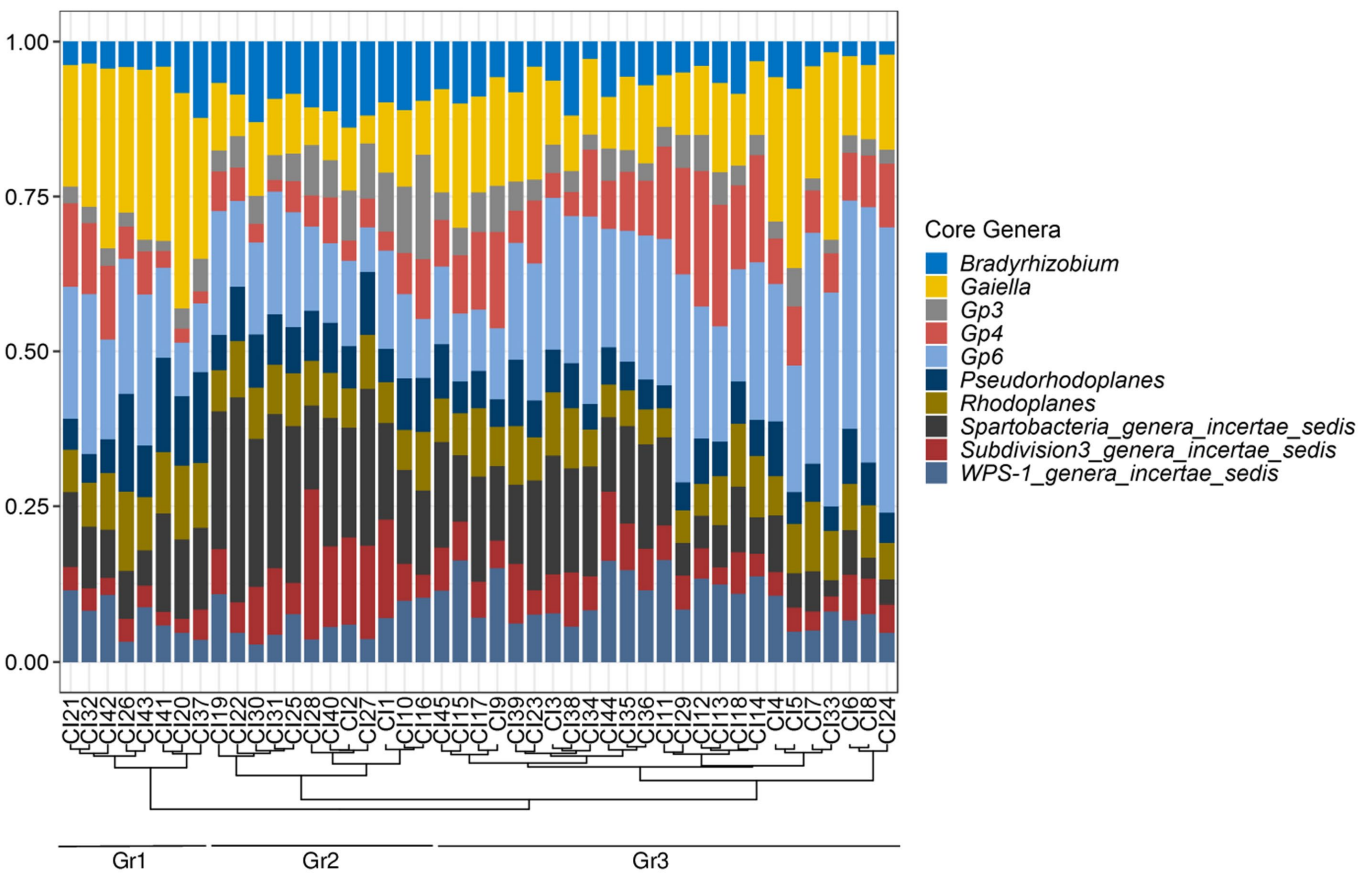
The core bacteriobiome of Côte d’Ivoire soils: taxonomic distribution per sample for the most abundant genera with 100% occurrence. Hierarchical clustering of samples based on the relative abundances of the core genera is shown by the dendrogram on the bottom of the plot which revealed three distinct groups: Gr1, Gr2, and Gr3.

The distribution of these genera in the 45 soil samples is represented in Figure 5. *Gp4* and *WPS-1_genera_incertae_sedis* were differently distributed in the soils of the three types of vegetation (*p* < 0.01), while the other genera were homogeneously distributed (*p* > 0.05) (Supplementary Table S6). A hierarchical clustering of the core genera abundance based on the Bray distance revealed three distinct groups of soils (Figure 5). The first group Gr1 (18% of soil samples) had a high abundance of *Gaiella* and low abundance of *Bradyrhizobium*, *Spartobacteria_genera_incertae_sedis* and *Subdivision3_genera_incertae_sedis*, respectively. The second group Gr2 (27%) had high abundance of *Bradyrhizobium*, *Spartobacteria_genera_incertae_sedis*, and *Subdivision3_genera_incertae_sedis*. The third group Gr3 (55%), was dominated by *Gp6* (Figure 5). The Wilcoxon rank sum test used showed that the *Gaiella* genus had different abundances between the three groups while *Bradyrhizobium, Gp3*, *Gp6 and Subdivision3_genera_incertae_sedis* were evenly distributed in both groups Gr1 and Gr3. Moreover, *Rhodoplanes* was equally present in both Gr2 and Gr3 groups. The abundance of *Pseudorhodoplanes* as well as *Gp4* was the same across the Gr1 and Gr2 groups, while the abundance of *WPS-1_genera_incertae_sedis* was found to be different in Gr2 and Gr3 groups (Supplementary Table S7).

### 3.5. Main variables influencing the core bacteriobiome distribution across Côte d’Ivoire soils

**Figure 6:**
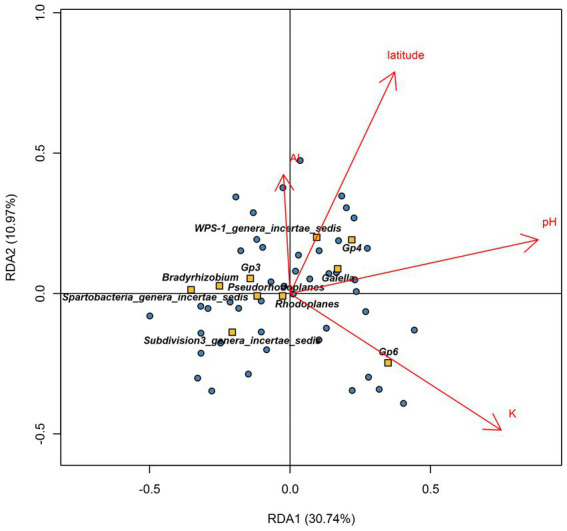
Redundancy analysis (RDA) ordination diagram (triplot) of core bacteria genera and physicochemical factors across Côte d’Ivoire soils. The plot shows samples in dots, explanatory physicochemical variables (red arrows), and the response variables (orange square with genera names). Axis 1 and axis 2 explained 37.9 and 9.6% of the total variation, respectively. Positively correlated variables are shown as arrows going in the same direction.

Redundancy analyses were used to identify the main factors that influenced the core bacteriobiome distribution. These factors included the pH (*R*^2^ = 22.85%), K (*R*^2^ = 9.35%), latitude (*R*^2^ = 5.56%) and Al (*R*^2^ = 2.98%) (Figure 6). Moreover, Spearman’s correlation coefficient analyses showed that alkaline pH positively affected *Gp4* abundance (*ρ* = 0.6, *p* < 1e-04) but not *Bradyrhizobium* (*ρ* = − 0.62, *p* < 1e-04) and *Spartobacteria_genera_incertae_sedis* (*ρ* = −0.59, *p* < 1e-04), that were more abundant in acidic soils. However, K-rich soils were shown to have an increased abundance of *Gp6* genus (*ρ* = 0.59, *p* < 1e-04). However, soils with high K concentrations had low abundance of *Bradyrhizobium* and *Gp3* (*Bradyrhizobium*: *ρ* = −0.6, *p* < 1e-04; Gp3: *ρ* = −0.65, *p* < 1e-04). Lastly, the abundance of *Gp4* (*ρ* = 0.52, *p* = 2e-04) and *WPS-1_genera_incertae_sedis* (*ρ* = 0.67, *p* < 1e-04) were highly correlated with increasing latitude values (Figure 6).

Interactions among genera belonging to the core bacteriobiome were also analyzed (Supplementary Table S8; Supplementary Figure S4). It was shown that the abundance of the genera *Bradyrhizobium*, *Gp3*, *Spartobacteria_genera_incertae_sedis*, *Subdivision3_genera_incertae_sedis* and *Pseudorhodoplanes* were positively correlated (*r* ≥ 0.5, *p* < 0.001). Soils with higher abundance of *Gp6* genus shows low abundance of *Bradyrhizobium* (*r* = −0.6, *p* < 0.001). The abundance of *Rhodoplanes* was strongly correlated to *Pseudorhodoplanes* (*r* = 0.8, *p* < 0.001) and *Gaiella* (*r* = 0.5, *p* < 0.001), whereas *WPS-1_genera_incertae_sedis* abundance was strongly correlated with *Gp4* (*r* = 0.7, *p* < 0.001).

### 3.6. Predicted functions of the core bacteriobiome in Côte d’Ivoire soils

**Figure 7:**
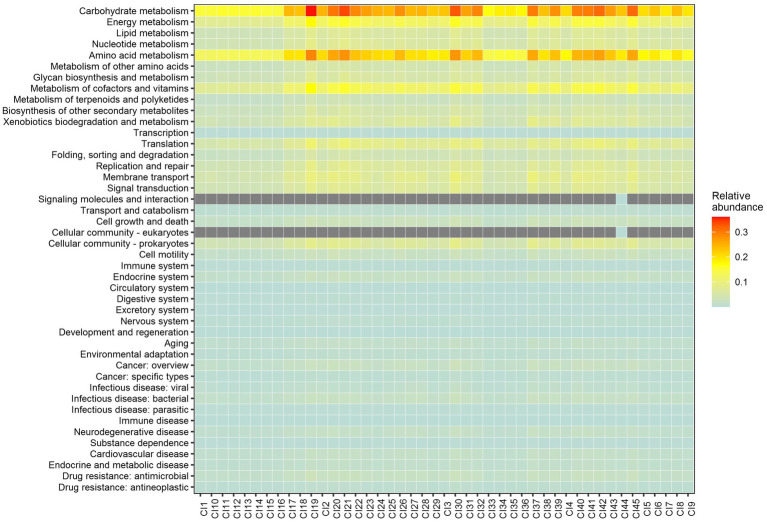
PICRUSt2 predicted metabolic capacities of the core genera based on 16S rRNA genes and the KEGG database. Abundance values are ranked from the lowest (light blue) to the highest (red). The grey boxes means no value recorded.

A total of 44 metabolic capacities were used to predict the functional properties of the core bacteriobiome. The most abundant predicted metabolic capacities were Carbohydrate Metabolism and Amino Acid metabolism (Figure 7).

**Figure 8:**
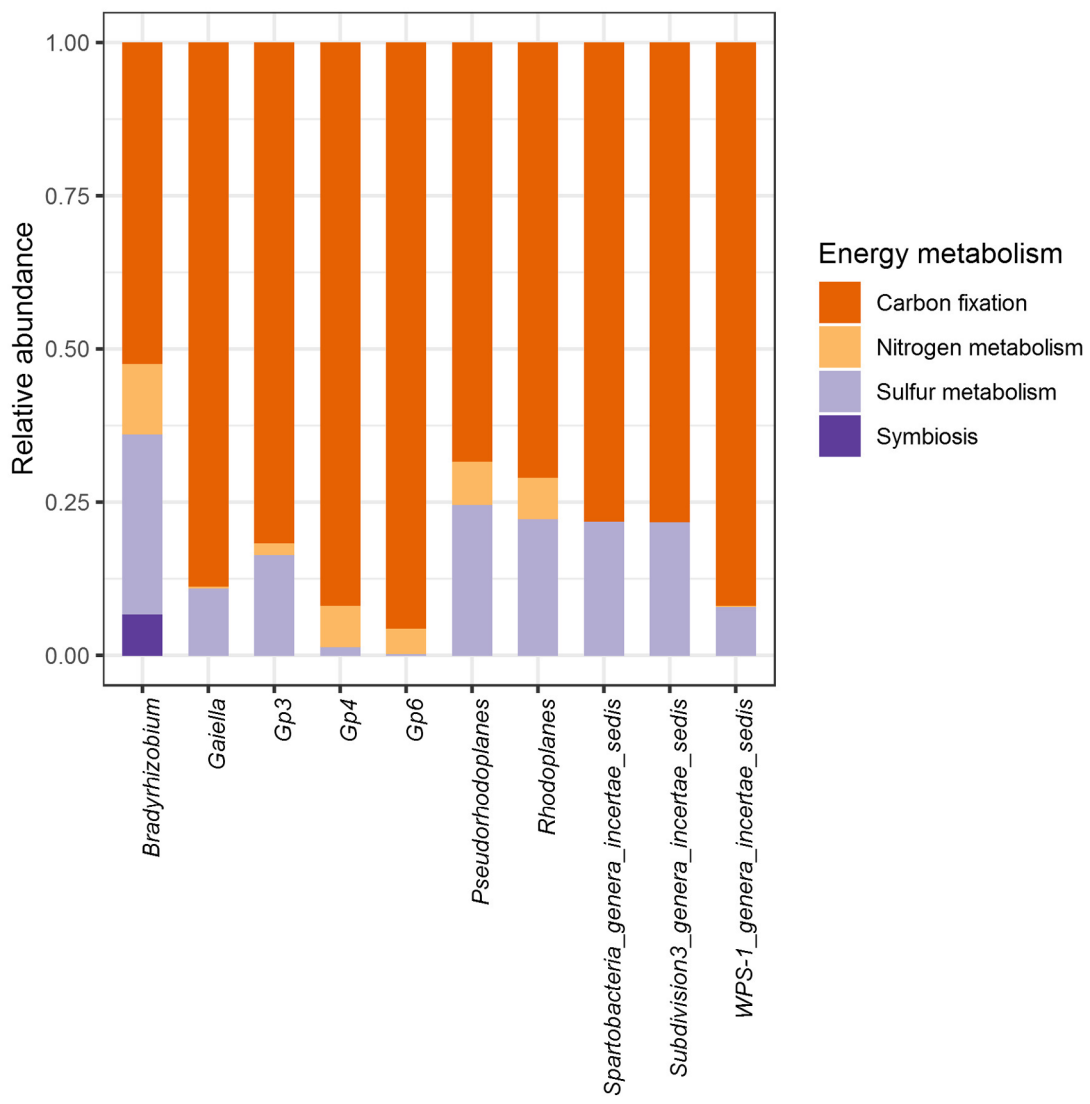
Relative abundance of genes associated with agriculturally important energy metabolism functions in the core bacteriobiome of Côte d’Ivoire soils.

Further predictive functional analyses were performed for all 10 dominant genera, focusing on energy metabolism, particularly the capacity for carbon, nitrogen and sulfur cycling. In general, it was shown that carbon, nitrogen and sulfur cycles were statistically different within each bacterial genus (Figure 8; Supplementary Table S9). In contrast, the analysis revealed that only sulfur metabolism and autotrophic carbon cycle were different according to soil samples (*p* < 2.2e-16) (Supplementary Figure S5). Carbon sequestration was the most abundantly predicted and was significantly different among vegetation type (*p* < 2.2e-16) just like sulfur metabolism (*p* = 3.8e-10). Nitrogen metabolism (*p* = 0.13) and symbiosis (*p* = 0.9) showed no significant difference through the three biomes.

In terms of occurrence, carbon sequestration was by far the most frequently predicted function, followed by sulfur metabolism, nitrogen metabolism. As for the nitrogen, symbiotic fixation was found to be possible in *Bradyrhizobium* only (Figure 8).

**Figure 9:**
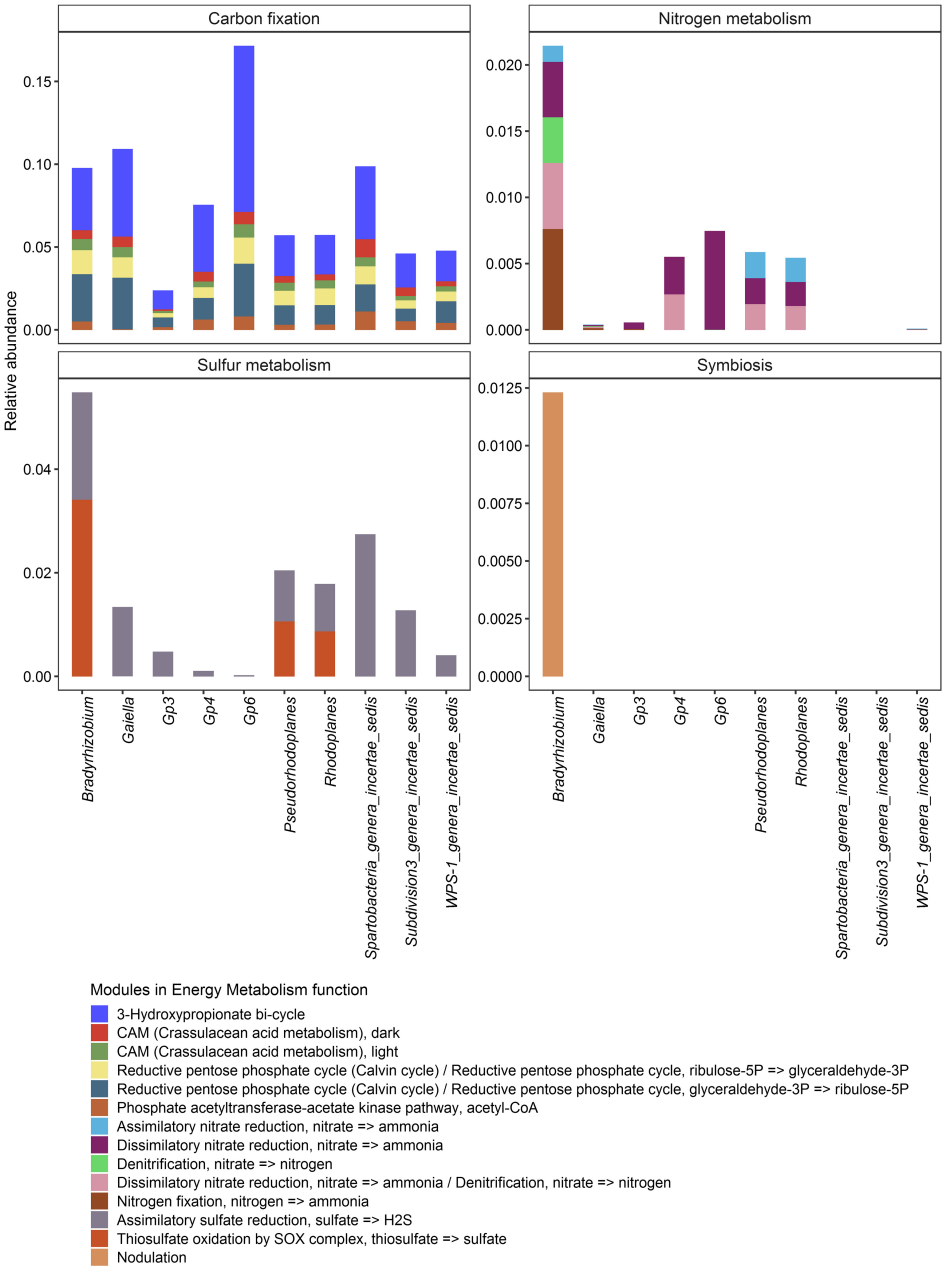
Distribution of module abundance within each energy metabolism function for each core genus. The plot was split regarding the main Energy metabolism functions. No bar means that there was no data for the concerned genus.

Interestingly, *Bradyrhizobium* was also the only genus from the core bacteriobiome capable of performing all the energy metabolism functions (Figure 9). All the 10 core genera potentially metabolized sulfate to hydrogen sulfide through assimilatory sulfate reduction, while only *Bradyrhizobium*, *Pseudorhodoplanes* and *Rhodoplanes* (Proteobacteria genera) are predicted to oxidize thiosulfate to sulfate (Figure 9; Supplementary Table S9).

## 4. Discussion

This work aimed to assess the landscape-scale distribution of bacteria in Côte d’Ivoire soils and characterize the environmental factors that may influence the structure of the core bacteriobiome. Studies of the biogeography of soil microorganisms have been performed at large scale worldwide (Griffiths et al., 2011; Malard et al., 2019) including, most recently, Sub-saharian Africa (Cowan et al., 2022). However, nationwide studies, as done elsewhere (Karimi et al., 2018), have not been performed in sub-Saharan Africa and the soil microbiomes remain unexplored in terms of key bacterial taxa abundance, prevalence and functions. It is particularly true for countries like Côte d’Ivoire, where few studies have been conducted on the field of soil bacterial ecology. Despite its large biome diversity, including fallow, gallery-forest, primary-forest, forest-savannah mosaic, shrubland and wooded-savannah landscapes (Tra Bi et al., 2015), soil chemical data suggested that Côte d’Ivoire soils properties were only partially dependent on biome type. For example, acidic soils were observed across all three major biomes. The significant differences observed among the three vegetation types concerning C and N contents and climate data were not surprising as it has been already reported elsewhere (Tondoh et al., 2015; Kassin et al., 2017).

In order to assess the bacterial distribution through the diverse vegetation types of Côte d’Ivoire soils, HTAS analysis of the 16S rRNA gene have been performed. The analysis revealed that Côte d’Ivoire soils were dominated in terms of abundance and prevalence by 10 bacterial phyla, including Acidobacteria, Actinobacteria, Bacteroidetes, candidate division WPS-1, Chloroflexi, Firmicutes, Gemmatimonadetes, Planctomycetes, Proteobacteria and Verrucomicrobia as reported elsewhere (Cowan et al., 2022). It is commonly accepted that these phyla are the ones most frequently found in the top layers of soils worldwide (Delgado-Baquerizo et al., 2018; Karimi et al., 2018). Here, the distribution of the 10 bacterial phyla was substantially driven by latitude, K content, clay, pH and Al content. pH has repeatedly been reported as one of the major predictors of soil bacterial community composition (Fierer and Jackson, 2006; Wei et al., 2017; Karimi et al., 2018; Liu et al., 2018; Cheng et al., 2020). It was shown that there was an inverse relationship between pH and Actinobacteria abundance where Actinobacteria were less abundant in the forest biome with lower pH while Acidobacteria were higher, as demonstrated elsewhere (Lauber et al., 2009).

It has been reported that different environmental factors can lead to differences in niches, resulting in the occurrence of a core microbiome (Turnbaugh et al., 2007). Furthermore, it has been argued that, the identification of the core microbiome is important since its persistent presence in particular habitats is likely to be essential for their functioning (Shade and Handelsman, 2012). However, despite its importance, there has been a little real consensus on how a core microbiome should be defined (Neu et al., 2021). Conditions in the determination of a core microbiome vary enormously, depending on the purpose of the study. It is often defined at different taxonomic levels, notably at the genus level (Lupwayi et al., 2021) or at the OTUs/ASVs level as reported in several studies (Gołębiewski et al., 2014; Jiao et al., 2019; Taye et al., 2020; Gschwend et al., 2021). Neu et al. (2021), reported three methods to quantify a core microbiome, for example by the occurrence in samples as in Gschwend et al. (2021), the relative abundance or by the combination of the occurrence and abundance. The latest method has been often used in recent studies (Jiao et al., 2019). Neu et al. (2021) also reported the wide variation in threshold value used to define the core microbiome, varying from 50 to 100% for occurrence, and 0.001–4.5% for abundance. Regarding all these different methods, this study choose to focus on a genus level core microbiome based on a combination of abundance and occurrence conditions. In an analysis of the core bacteriobiome of Côte d’Ivoire soils, a total of 10 genera belonging to the 10 major phyla were found to be prevalent in at least 95% of all soils with an abundance of above 0.5%. These were *Bradyrhizobium*, *Gaiella*, *Gp3, Gp4, Gp6, Pseudorhodoplanes, Rhodoplanes, Spartobacteria_genera_incertae_sedis, Subdivision3_genera_incertae_sedis and WPS-1_genera_incertae_sedis*. These taxa are known to be soil-associated (Navarrete et al., 2013; Gołębiewski et al., 2014; Taye et al., 2020; Lupwayi et al., 2021). The ubiquity of these genera may suggest an involvement in soil structure and functioning, or may serve as an indicator of soil quality or be linked to a particular vegetation/crop type (Simonin et al., 2020). For example, members of *Bradyrhizobium* genus, known to be able to establish symbioses with leguminous plants (Woomer et al., 1988; Bouznif et al., 2019), were recently found to dominate in tropical savannah soils of Côte d’Ivoire in a legume cropping area (Gnangui et al., 2021). The *Gaiella* genus may have an important role in agricultural soils as a plant decomposer and inhibitor of *Fusarium oxysporum f*. sp. *Lycopersici* in tomato fields (Zhao et al., 2019). This functional trait in *Gaiella* may be of importance for Côte d’Ivoire, where vegetable-based agriculture are increasing (Soro et al., 2008). Along with *Gp4* and *Gp6*, the predominance of *Gp3* (Navarrete et al., 2013) could potentially be linked to the wide range of agricultural activities (Dedysh and Sinninghe Damsté, 2018), since more than 60% of the soils sampled were recovered from agricultural regions (see BioProject PRJNA695288). The high abundance of genus *Spartobacteria_genera_incertae_sedis* in Côte d’Ivoire soils was consistent with previous studies (Brewer et al., 2016) which have demonstrated that this taxon is ubiquitous and abundant in many soils types as grassland. It was evident that in addition to the (weak) influence of vegetation type, there were a combination of different variables that primarily shaped the distribution of the core bacteriobiome in Côte d’Ivoire soils. For example, *Gp6*, *Gp4* and *WPS-1_genera_incertae_sedis* were abundant in soils with highest values of pH, K, Ca, Mn and lower rainfall (Navarrete et al., 2013). *Gaiella*, *Rhodoplanes* and *Pseudohodoplanes* were abundant in soils with a pH level of about 6.1, with an intermediate content of K, Ca, Mn, Fe in biomes with moderately high rainfall level, being consistent with previous reports (Hiraishi and Ueda, 1994; Albuquerque et al., 2011; Supplementary Tables S7, S10). The fact that *Bradyrhizobium* and *Gp3* abundances were also linked to pH levels further confirms the involvement of this parameter in bacterial communities structuration as previously reported (Woomer et al., 1988; Kielak et al., 2016; Dedysh and Sinninghe Damsté, 2018).

Core microbial taxa are thought to be of importance in agroecosystem servicing (Risely, 2020). Most of the predicted functions for *Gp6* were related to carbon metabolism (more than 80%), as for *Gaiella*, *Gp3*, *Gp4* and *WPS- 1_genera_incertae_sedis*. The other genera, on the other hand, shown the ability to vary their source of mineral resources by less carbon fixation (ranging from about 75–50% for *Bradyrhizobium* only) and the use of nitrogen and/or sulfur. The significant difference (*p* < 0.05) in the sulfur metabolism across soils could reveal that depending on the chemical properties of the environment (sulfur availability for example) certain species would be able to perform such oxidation. Unsurprisingly, only members of the Proteobacteria (*Bradyrhizobium*, *Rhodoplanes*, and *Pseudorhodoplanes*) had the putative ability to oxidise thiosulfate, a key sulfur cycle compound, into sulfate (Zhang et al., 2020). Another important predicted function is related to nitrogen metabolism mainly performed by *Bradyrhizobum*. In general important findings have been reported on the benefits of the microbial activities linked to N cycling, including N_2_ fixation, mineralization, nitrification and denitrification in Côte d’Ivoire soils. Culture-dependent studies have revealed that the genus *Bradyrhizobium* represents a source of potential bioinoculants capable of fostering the growth of diverse leguminous crops (Fossou et al., 2020). Recently, a pioneering study of soil bacteriobiome carried out in fields of central Côte d’Ivoire has shown that among the key microbial activities linked to N cycling, nitrification plays a crucial role in determining the outcome of agronomic practices (Assémien et al., 2017). Taken together, this exploratory work, as well as previous reports, provided novel insights into understanding the genetic and functional diversity of the key bacterial taxa in Côte d’Ivoire soils, and may serve as indicators for future microbiome explorations and for land-use decision-making in Côte d’Ivoire.

## 5. Conclusion

This work did a national scale metagenomic study in Côte d’Ivoire in order to investigate the bacterial communities inhabiting the soils sampled in several biomes. As a result, the bacterial population mainly encountered in these soils were unsurprisingly those that were commonly found in soils across the world with a main pH-based distribution. When assessing the core bacteriobiome, the major distribution scheme was found to be vegetation-independent. While the predominance of *Bradyrhizobium* genus in soils is well-known and documented, some other members of the core bacteriobiome such as *Gaiella* lack key information that could help in better understanding their role in Côte d’Ivoire soils. At genus level, *Bradyrhizobium* was the only taxon from the core bacterial microbiome capable of performing all the energy metabolism functions investigated, in addition to its capacity for nodulation through symbiosis. In sum, this work, which is the one of the pioneering study to ever be undertaken at this scale in Côte d’Ivoire, unraveled the complexity of the Côte d’Ivoire soil bacteriobiome, and opened a new era to investigate in deep its functions and services in the national ecosystem functioning.

## Data availability statement

The datasets presented in this study can be found in online repositories. The names of the repository/repositories and accession number(s) can be found at: https://www.ncbi.nlm.nih.gov/, PRJNA695288.

## Author contributions

DC and AZ: study conception, supervision, and project administration. YB, DV, and CA: data acquisition. DC, AZ, CA, and RF: methodology. CA, AE, DK, RF, and CK: software. CA and RF: validation and data curation. CA, AE, and RF: data analysis. CA: writing-original draft preparation. AZ, RF, CA, and AE: writing-review and editing. DC: funding acquisition. All authors read and approved the final manuscript.

## Funding

This research was funded by the US Agency for International Development (USAID). The grant number is 674-AA-2010-A1.

## Conflict of interest

The authors declare that the research was conducted in the absence of any commercial or financial relationships that could be construed as a potential conflict of interest.

## Publisher’s note

All claims expressed in this article are solely those of the authors and do not necessarily represent those of their affiliated organizations, or those of the publisher, the editors and the reviewers. Any product that may be evaluated in this article, or claim that may be made by its manufacturer, is not guaranteed or endorsed by the publisher.

## Footnotes

^1^ https://sourceforge.net/projects/rdp-classifier

^2^ http://hmmer.org/

^3^ https://www.kegg.jp
