# Supplementary material for: The core bacteriobiome of Côte d’Ivoire soils across three vegetation zones
Source: Front Microbiol. 2023 Aug 24;14:1220655. doi: 10.3389/fmicb.2023.1220655 (PMC10483230; doi:10.3389/fmicb.2023.1220655)
Supplement: Supplementary file 1 [file Data_Sheet_1.PDF]

The core bacteriobiome of Côte d'Ivoire soils across three vegetation zones

Chiguié Estelle Raïssa Amon[1*], Romain Kouakou Fossou[1], Anicet Ebou[1], Dominique Kadio Koua[1], Claude Ghislaine Kouadjo[2], Yao Casimir Brou[1], Don Rodrigue Rosin Voko Bi[3], Don A. Cowan[4] and Adolphe Zézé[1]

*** Correspondence:**
Chiguié Estelle Raïssa Amon*****
emmamon9@gmail.com

1. Supplementary Figures and Supplementary Tables

1.1. Supplementary Figures

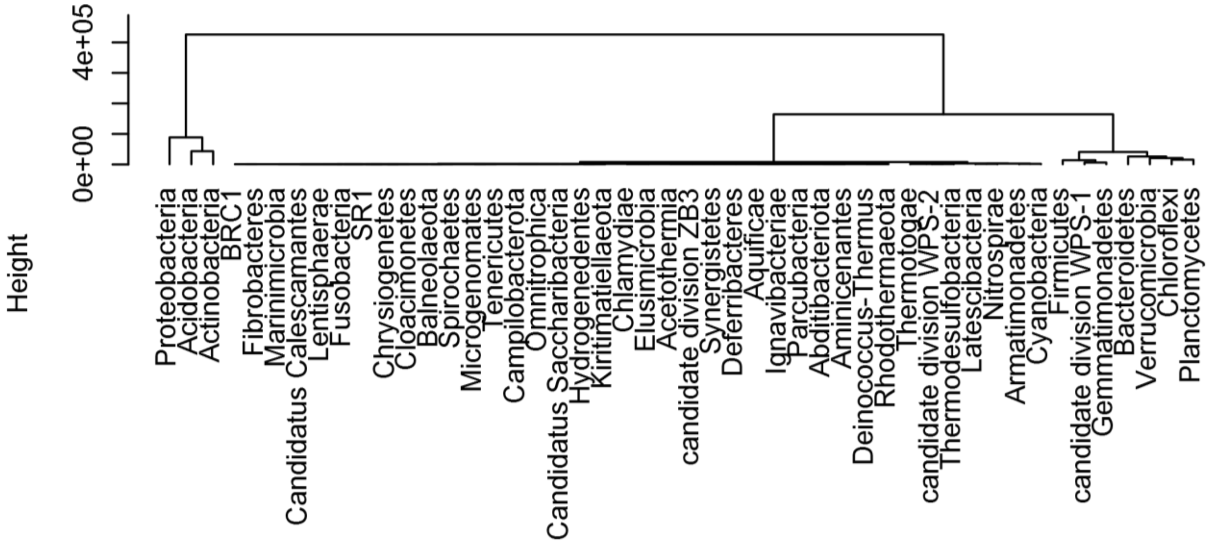

Supplementary Figure S1. Classification of phyla according to their abundance. The ascending hierarchical classification shows two distinct group.

A. Proteobacteria

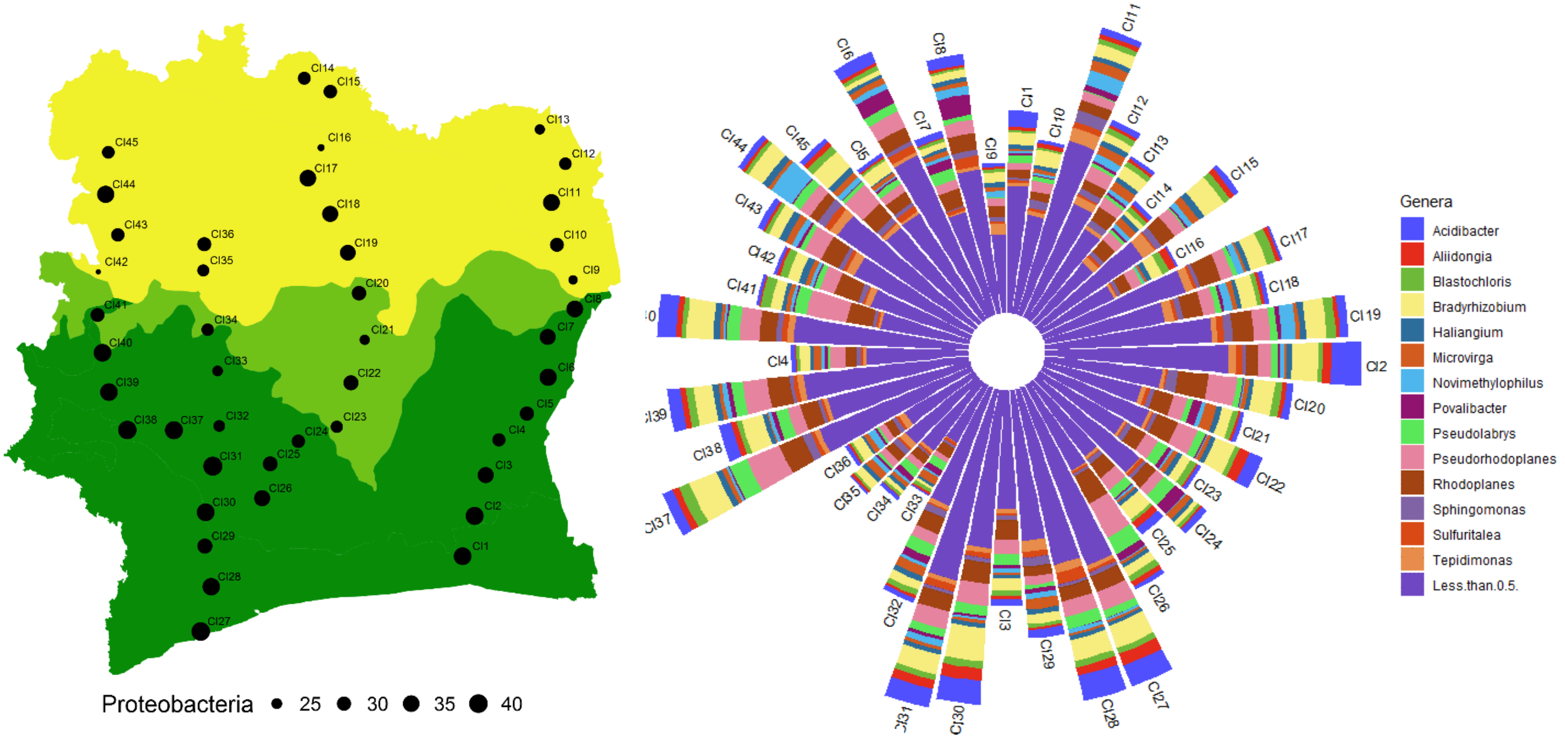

B. Actinobacteria

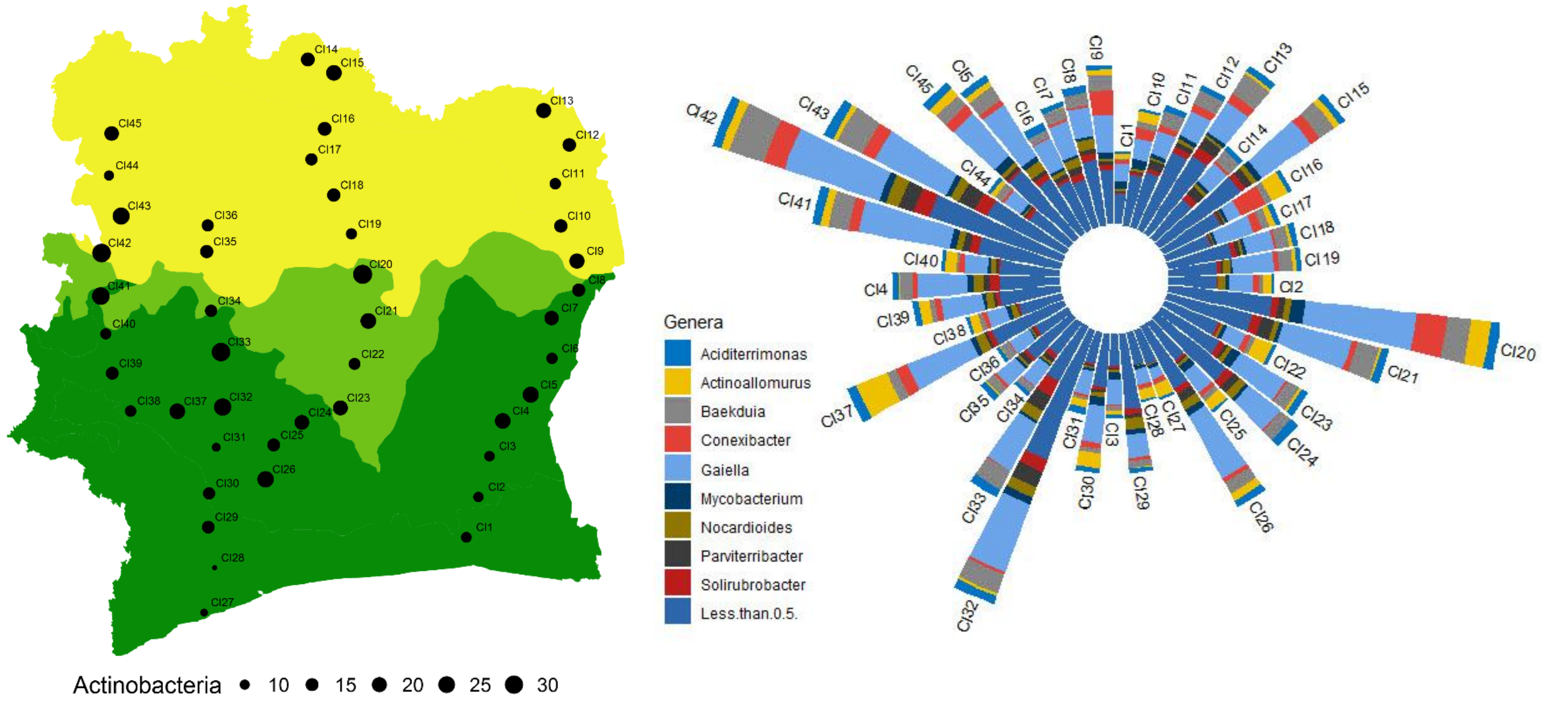

C. Acidobacteria

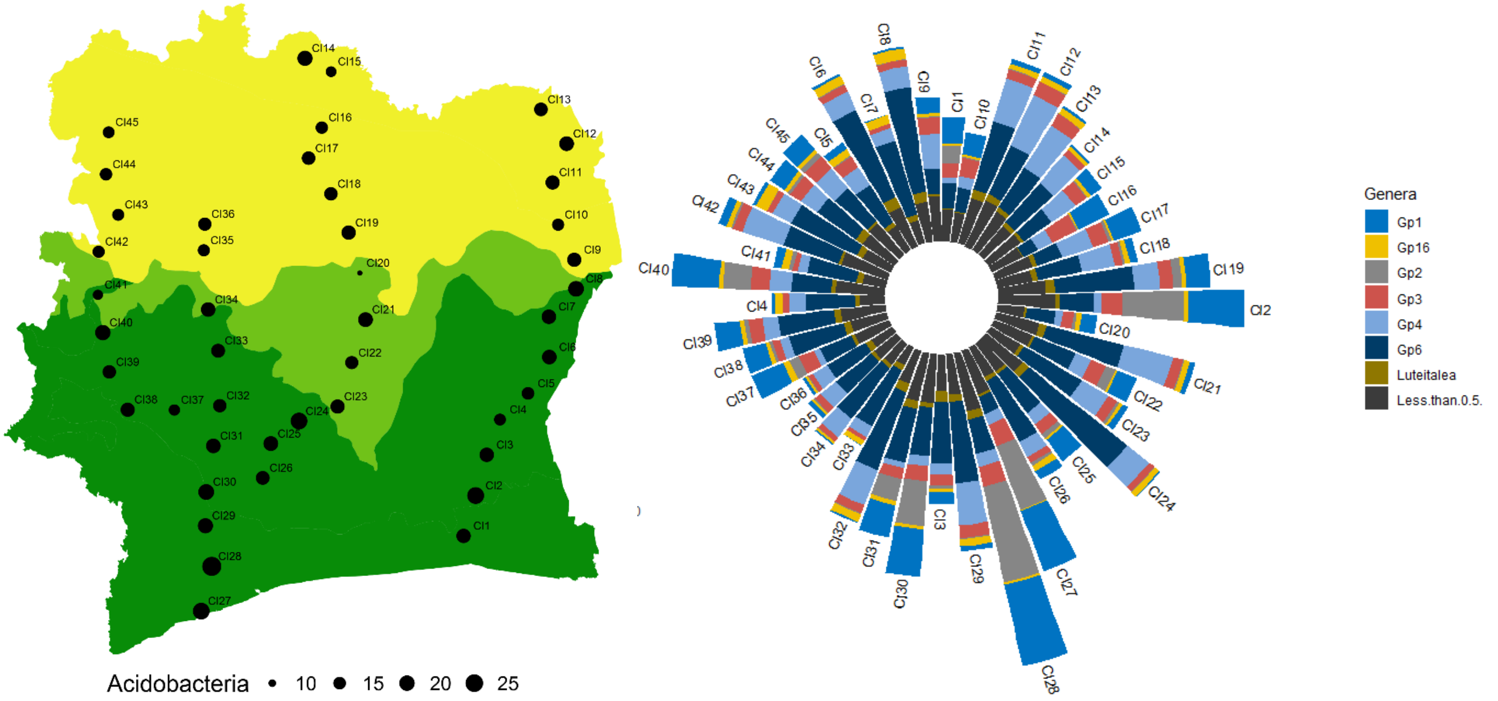

D. Bacteroidetes

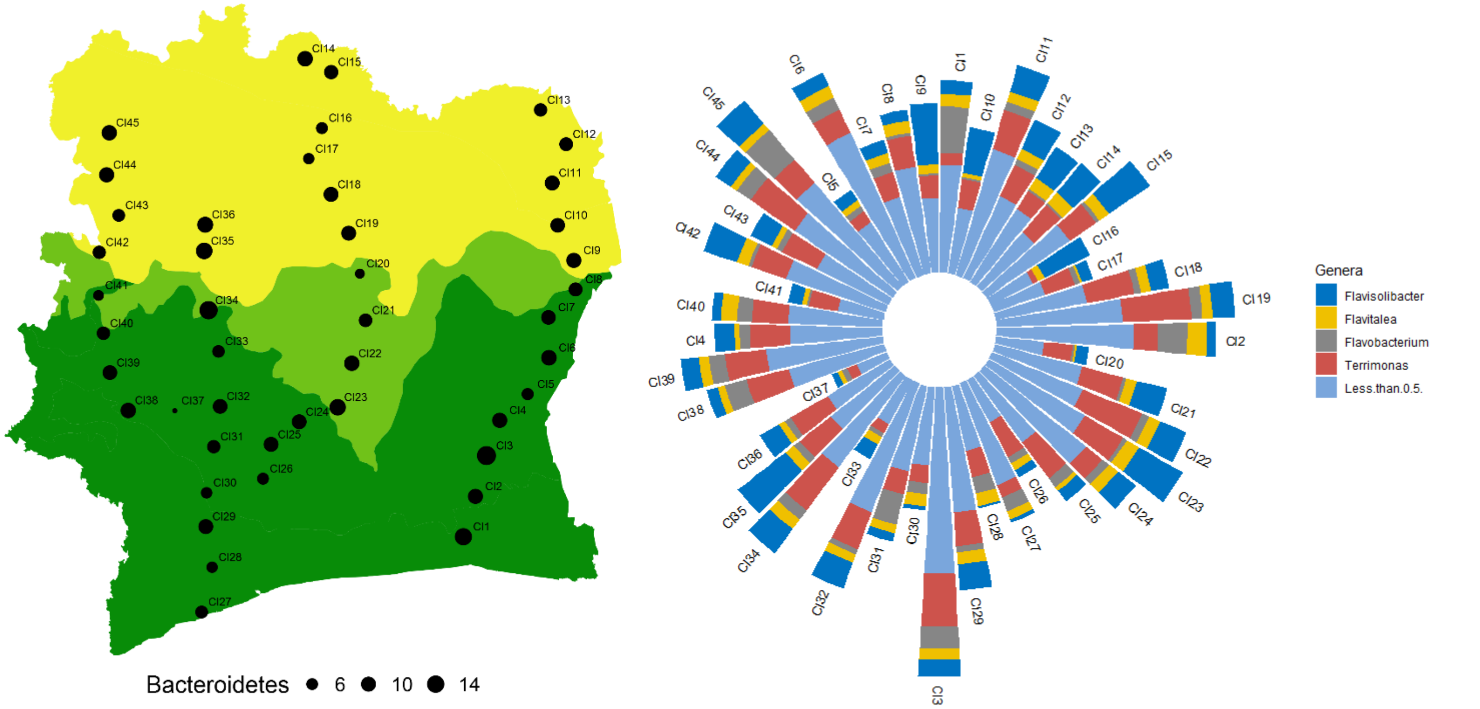

E. Verrucomicrobia

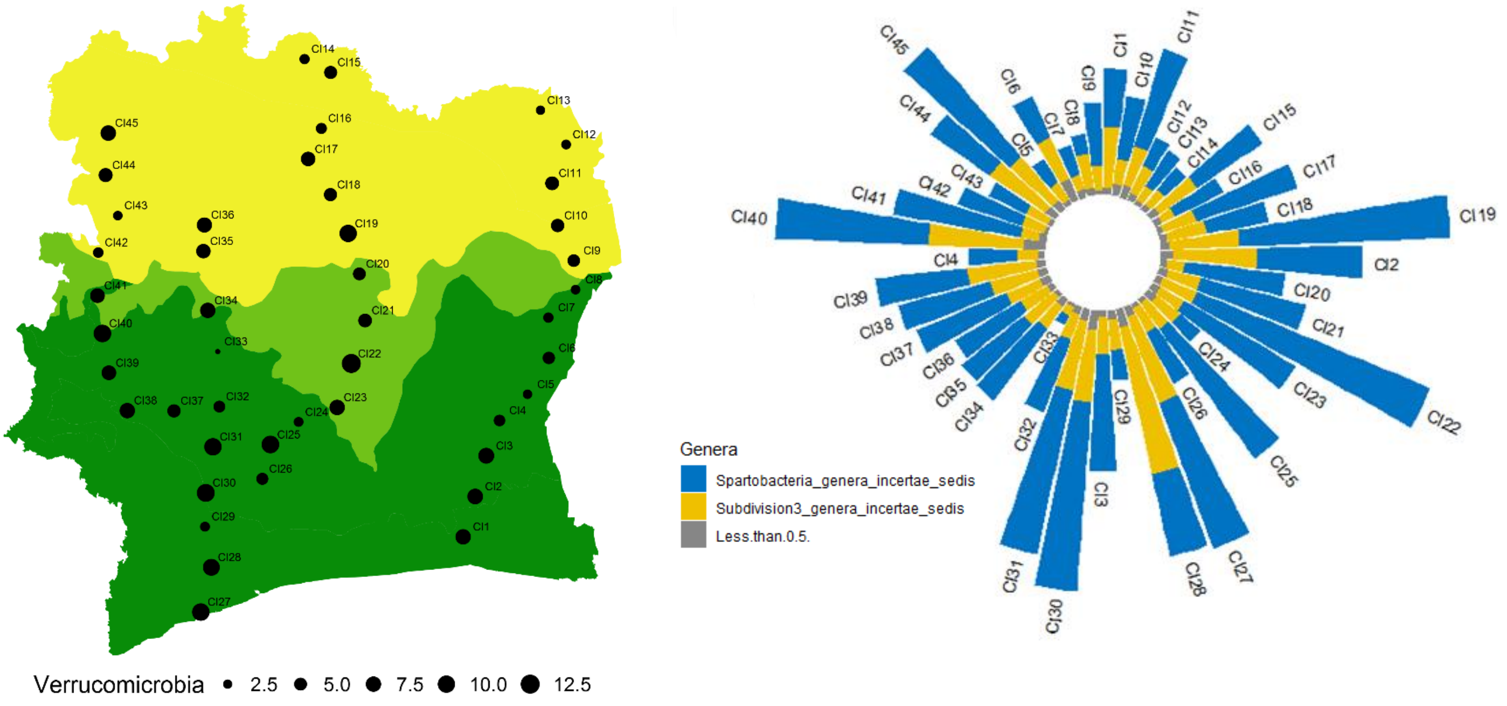

F. Planctomycetes

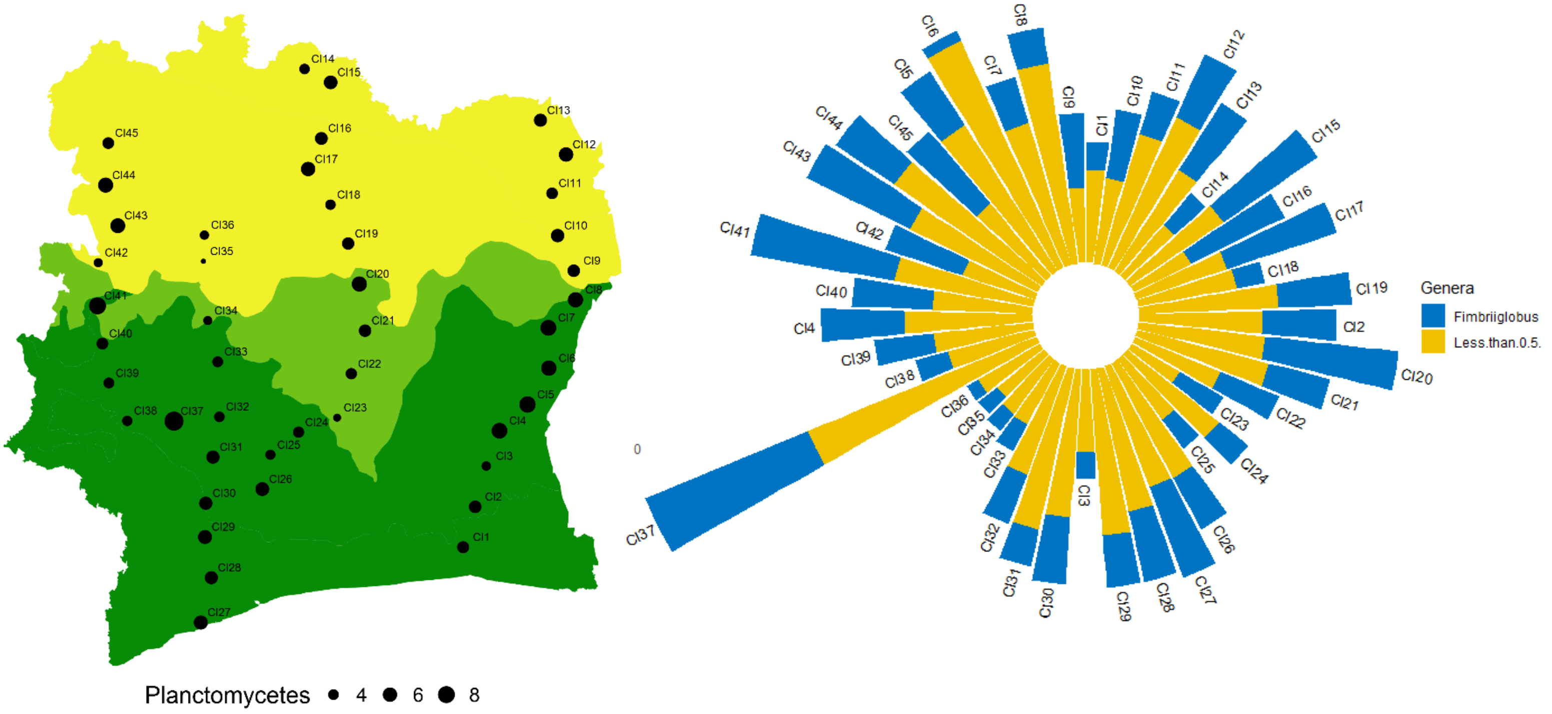

G. Chloroflexi

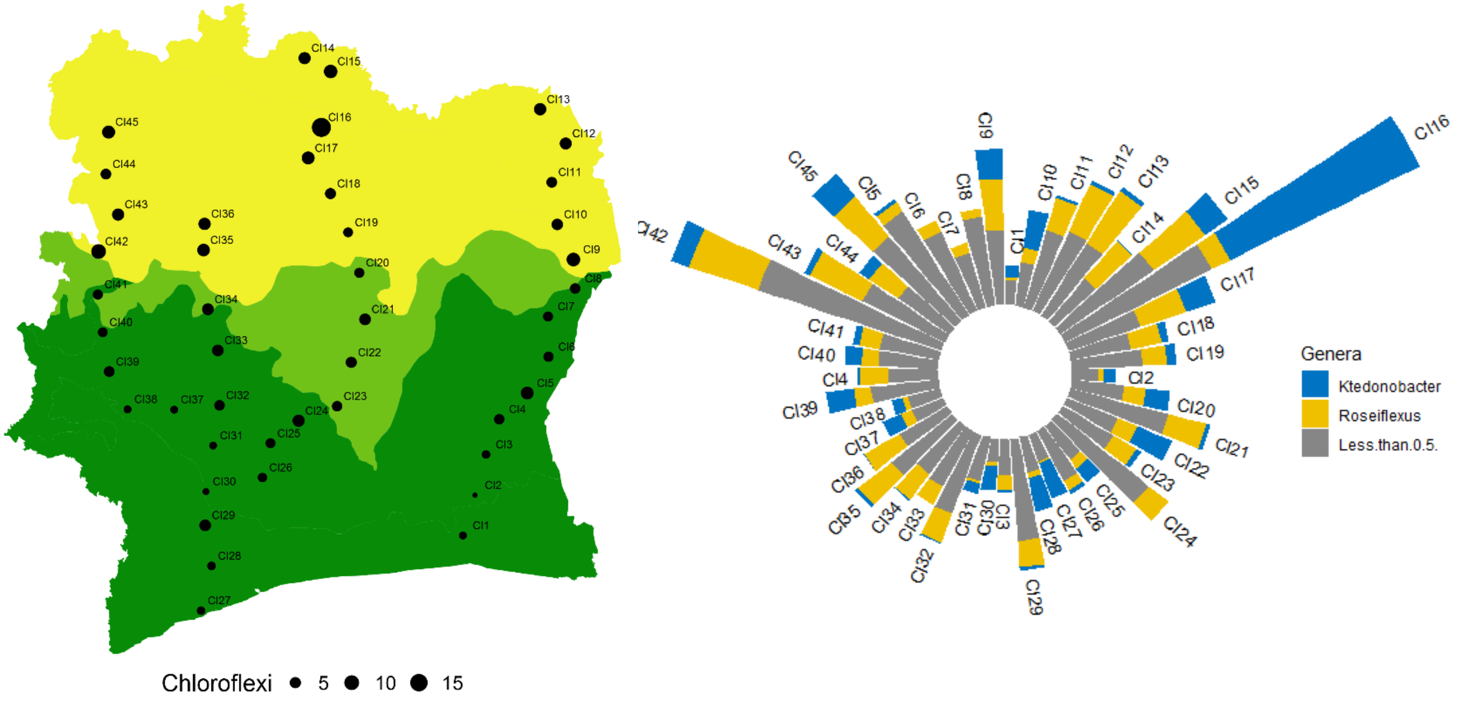

H. Firmicutes

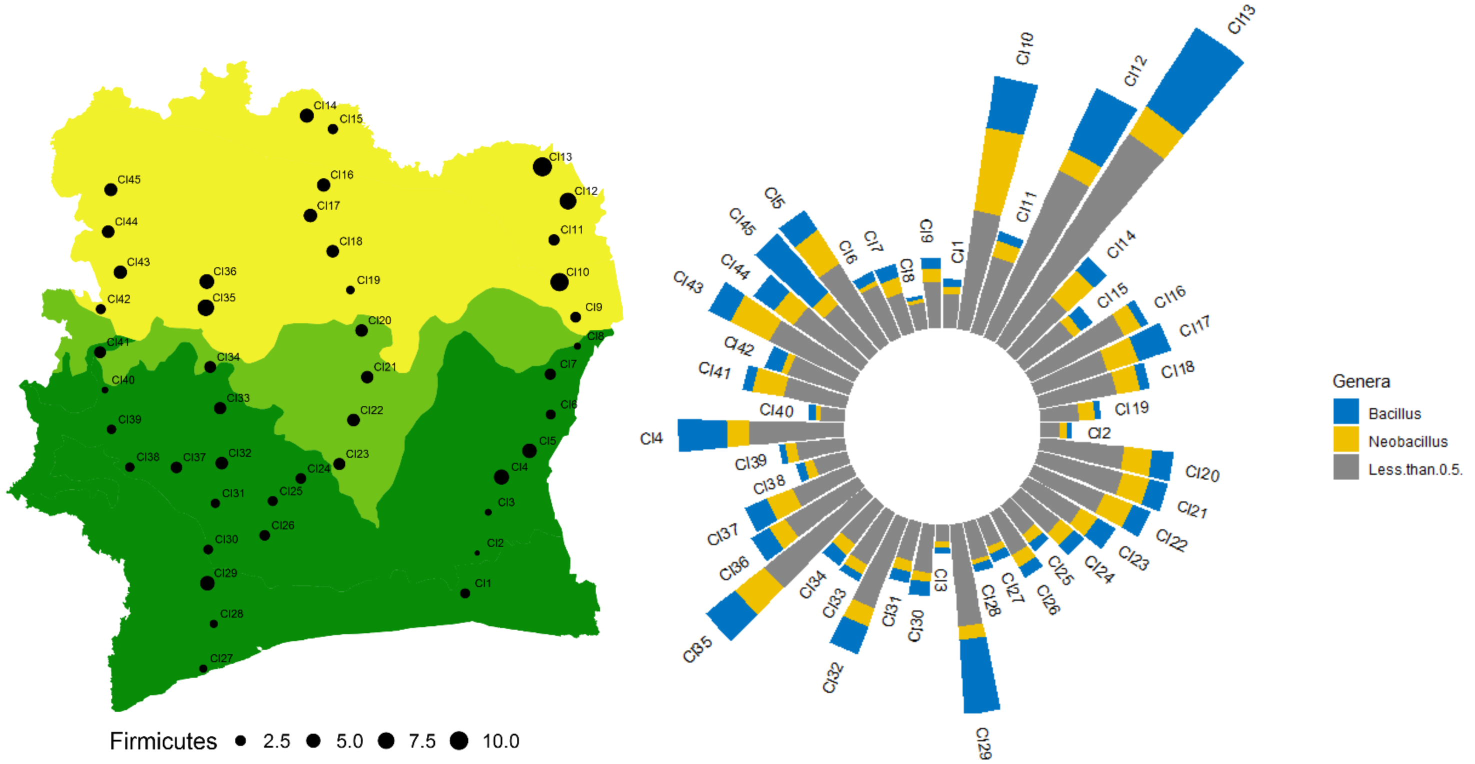

I. Candidate division WPS-1

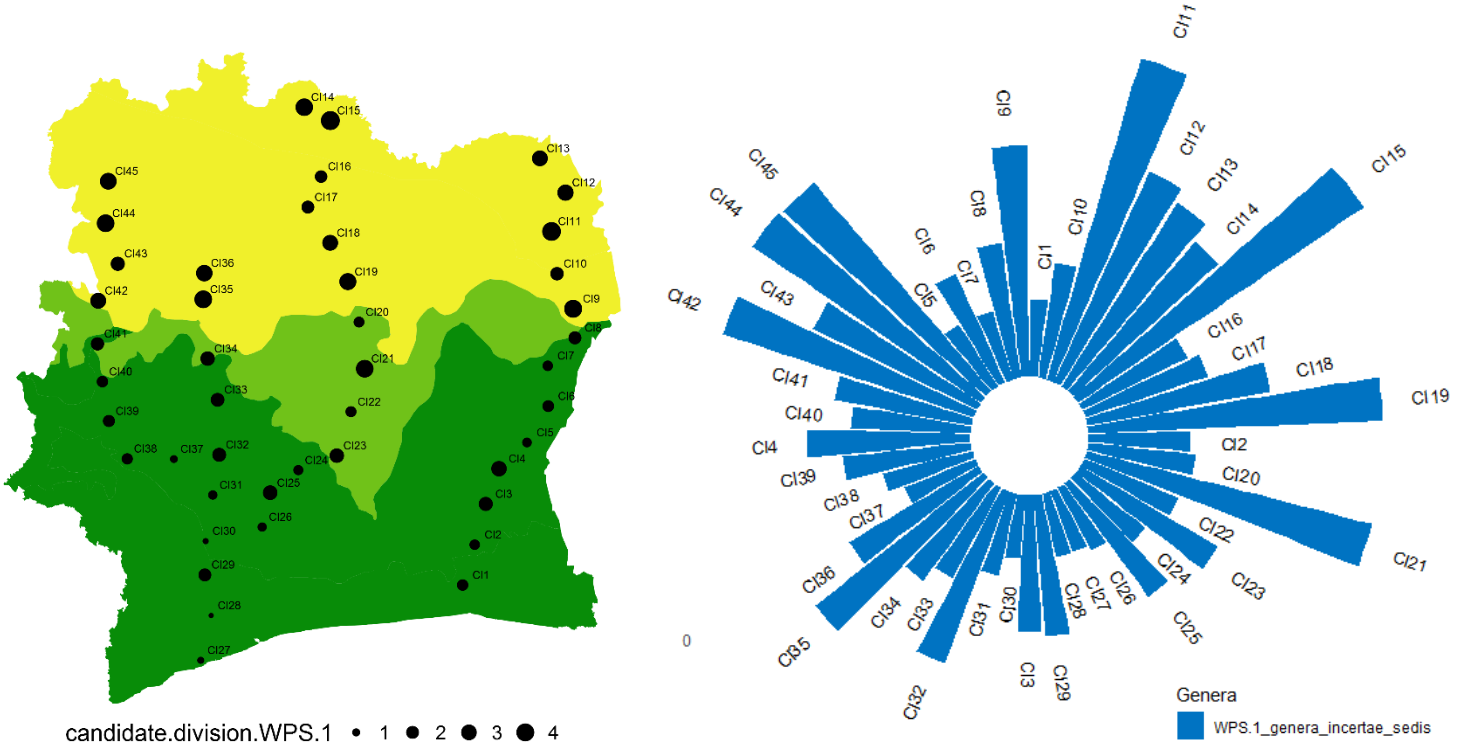

J. Gemmatimonadetes

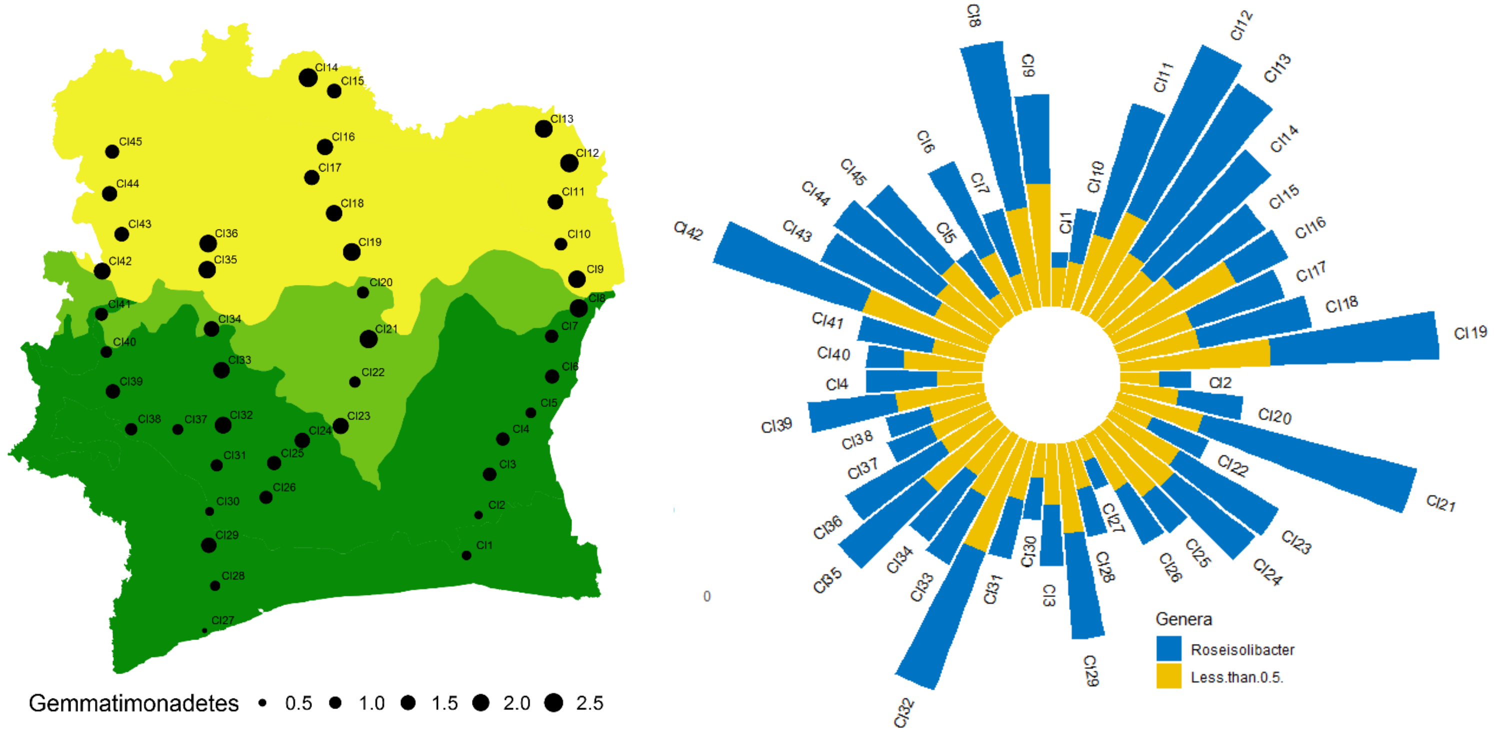

Supplementary Figure S2. Mapping of the major phyla in each soil sample based on their abundances. The maps are arranged from the most to the least abundant phyla and the size of the dots is a function of abundance. (On left) Maps of each phylum. (On right) Abundance of major genera (relative abundance > 0.5%) within each major phyla. Maps are colored according to the main vegetation types, from south to north: forest zone, forest-savannah contact zone and savannah zone.

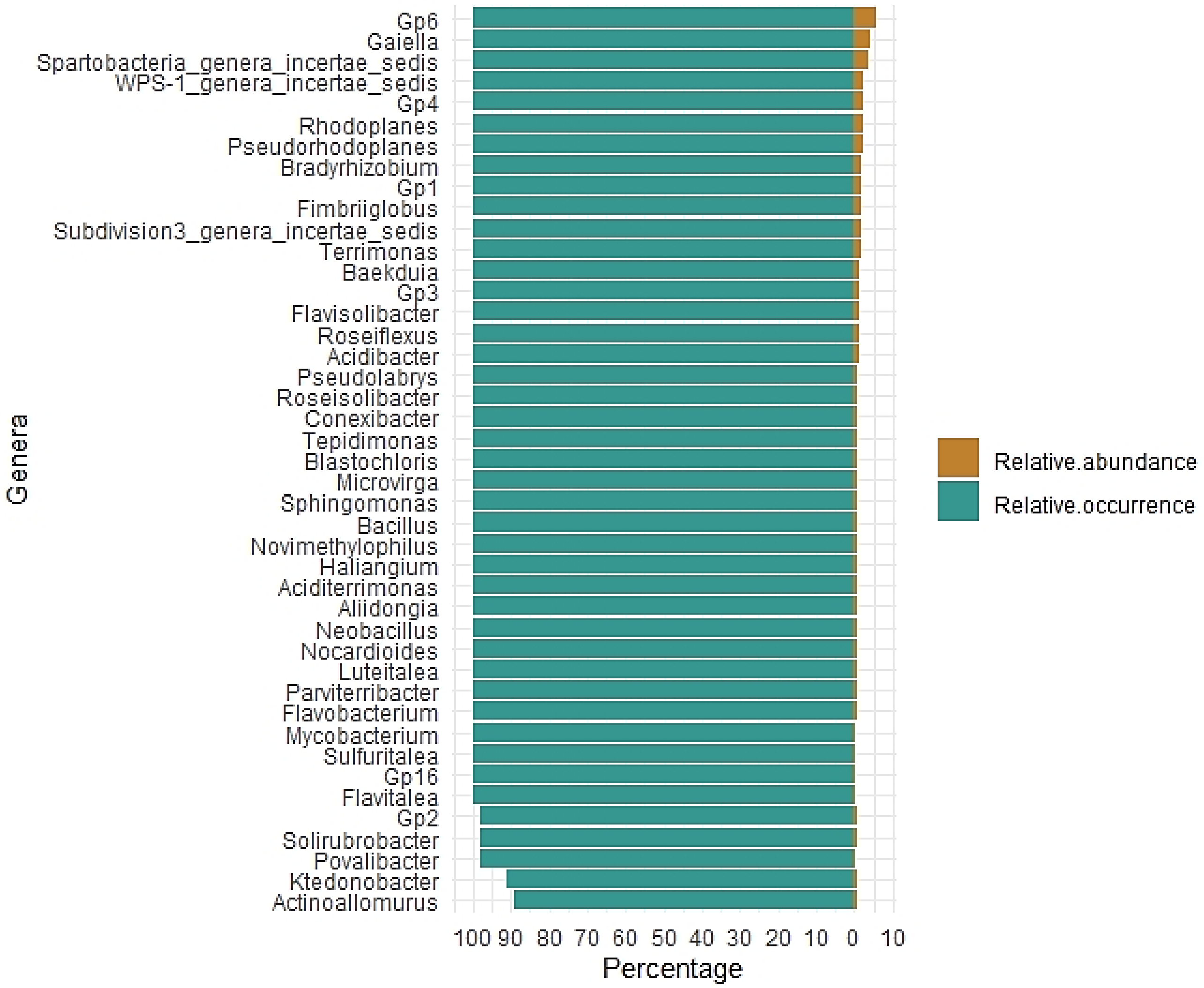

Supplementary Figure S3. Classification of the main bacterial genera from Côte d'Ivoire soils according to their abundance and relative occurrence. The genera are classified according to their relative occurrence. The occurrence varies from 100% for a total occupation of the soils to more than 80% for a presence in 40 soils. The most distributed and numerous genera have a relative abundance between 0.5 and 5.64%.

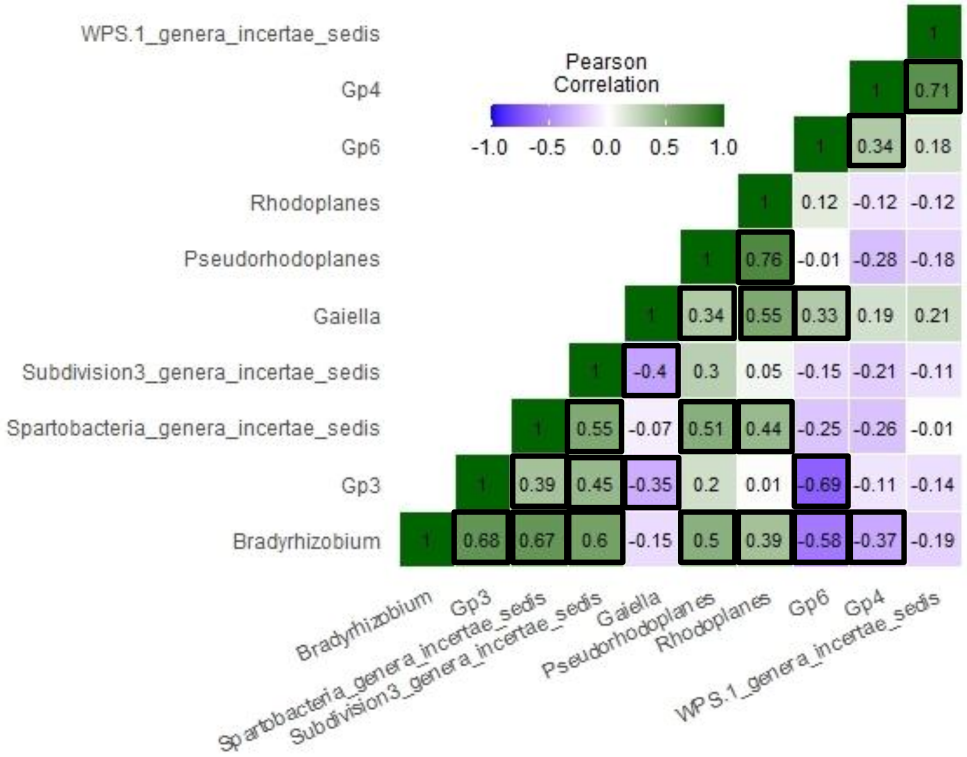

Supplementary Figure S4. Correlogram of the matrix of Spearman's rank order correlation coefficient among the different bacterial core genera. The positive correlation coefficient values are in green and the negative one in blue. Significant correlation are highlighted by squares with black borders.

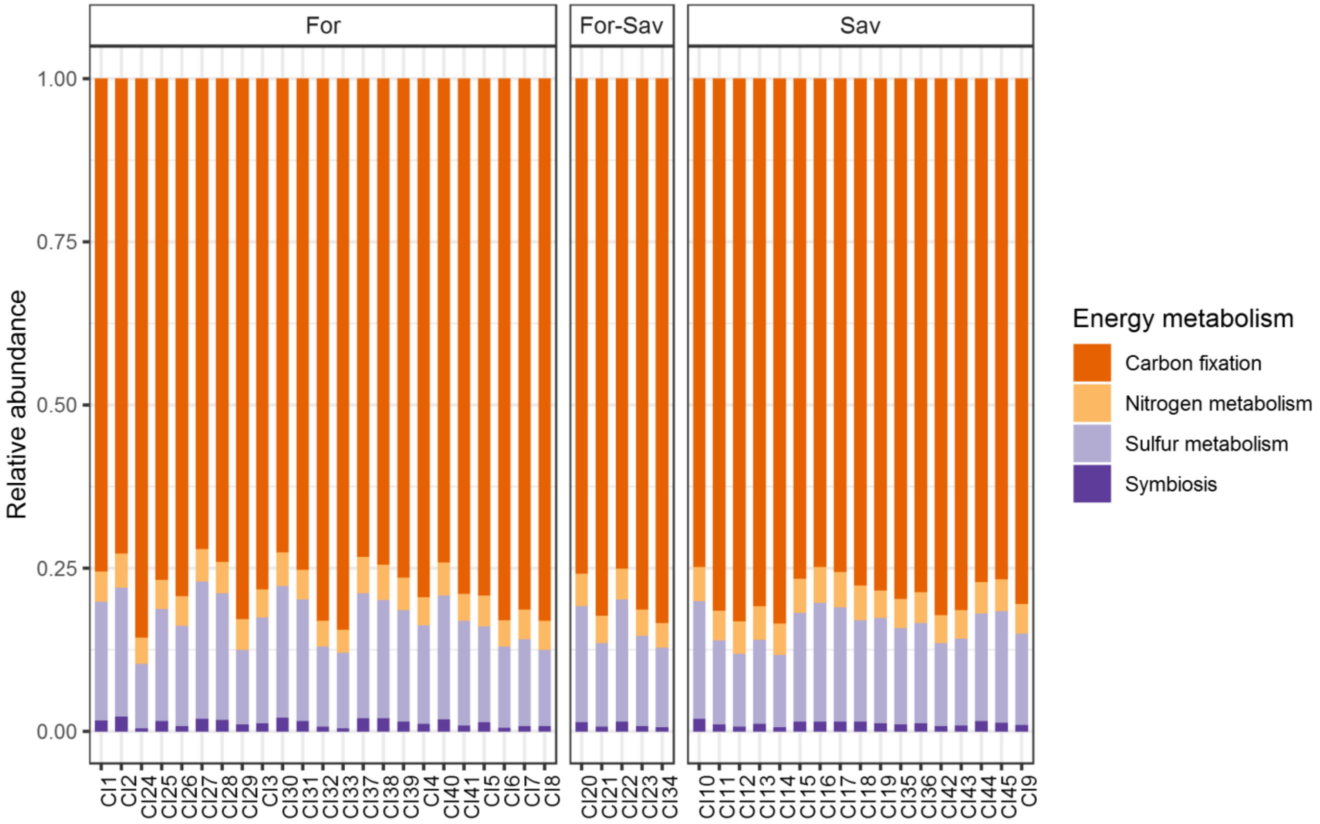

Supplementary Figure S5. Energy metabolism functions that may occurred through samples. The plot was generated by samples and split by vegetation types. For=forest zone, For-Sav=forest-savannah contact zone and Sav=savannah zone.

1.2. Supplementary Tables

Supplementary Table S1. Characteristics of sampling locations. Longitude and latitude are in decimal degrees (DD). Data includes information about the soils type according to the pH range: strongly acidic (pH<5.5), acidic (5.5 <pH<6.5), neutral (6.5<pH<7.5) and alkaline (pH>7.5) and the soils texture based on the USDA classification. SaLo=sandy loam, SaClLo=sandy clay loam, LoSa=loamy sand and Lo=loam

Samples	Localities	Longitude	Latitude	Vegetation Type	Soil type by pH	Soil texture
CI1	Anyama	-4,06	5,52	Forest Zone	acidic	SaLo
CI10	Tefrodouo	-3,13	8,57	Savannah Zone	acidic	LoSa
CI11	Bouna	-3,18	8,98	Savannah Zone	neutral	SaLo
CI12	Panzrani	-3,04	9,36	Savannah Zone	acidic	LoSa
CI13	Doropo	-3,30	9,70	Savannah Zone	neutral	LoSa
CI14	Ouangolo	-5,62	10,20	Savannah Zone	acidic	SaLo
CI15	Ferké	-5,37	10,07	Savannah Zon	neutral	SaLo
CI16	Sinematiali	-5,46	9,52	Savannah Zone	acidic	SaClLo
CI17	Kalogokaha	-5,59	9,22	Savannah Zone	acidic	SaLo
CI18	Kanawolo	-5,37	8,87	Savannah Zone	acidic	SaLo
CI19	Niakara	-5,19	8,49	Savannah Zone	strongly acidic	Lo
CI2	Adzopé	-3,94	5,91	Forest Zone	acidic	SaLo
CI20	Katiola	-5,08	8,09	Forest-Savannah contact zone	acidic	SaLo
CI21	Bouaké	-5,03	7,64	Forest-Savannah contact zone	neutral	SaLo
CI22	Tiebissou	-5,16	7,22	Forest-Savannah contact zone	acidic	SaClLo
CI23	Yamoussoukro	-5,30	6,79	Forest-Savannah contact zone	acidic	SaClLo
CI24	Kononfla	-5,68	6,64	Forest Zone	neutral	SaClLo
CI25	Ouragahio	-5,96	6,42	Forest Zone	strongly acidic	SaClLo
CI26	Gagnoa	-6,04	6,09	Forest Zone	acidic	SaClLo
CI27	San-Pédro	-6,65	4,78	Forest Zone	acidic	SaLo
CI28	Gabiadji	-6,55	5,22	Forest Zone	strongly acidic	SaLo
CI29	Méagui	-6,61	5,62	Forest Zone	neutral	LoSa
CI3	Akoupé	-3,83	6,31	Forest Zone	acidic	Lo
CI30	Soubré	-6,60	5,95	Forest Zone	acidic	SaLo
CI31	Issia	-6,53	6,40	Forest Zone	strongly acidic	SaLo
CI32	Zakoua	-6,47	6,79	Forest Zone	acidic	SaClLo
CI33	Vavoua	-6,48	7,33	Forest Zone	neutral	SaClLo
CI34	Alladjekro	-6,58	7,74	Forest-Savannah contact zone	neutral	SaClLo
CI35	Séguéla	-6,62	8,32	Savannah Zone	acidic	SaLo
CI36	Kani	-6,61	8,57	Savannah Zone	acidic	SaLo
CI37	Daloa	-6,91	6,75	Forest Zone	acidic	SaLo
CI38	Duekoue	-7,38	6,75	Forest Zone	strongly acidic	SaClLo
CI39	Logoualé	-7,56	7,13	Forest Zone	acidic	SaClLo
CI4	Abengourou	-3,70	6,66	Forest Zone	acidic	LoSa
CI40	Biankouman	-7,62	7,51	Forest Zone	acidic	SaClLo
CI41	Foungbégo	-7,67	7,88	Forest Zone	acidic	SaClLo
CI42	Touba	-7,66	8,30	Savannah Zone	acidic	SaClLo
CI43	Koro	-7,47	8,66	Savannah Zone	acidic	SaLo
CI44	Bako	-7,59	9,06	Savannah Zone	acidic	LoSa
CI45	Odienné	-7,56	9,47	Savannah Zone	acidic	SaLo
CI5	Zamaka	-3,42	6,92	Forest Zone	alkaline	SaLo
CI6	Koun-fao	-3,21	7,27	Forest Zone	neutral	SaLo
CI7	Tanda	-3,22	7,67	Forest Zone	acidic	Lo
CI8	Bondoukou	-2,95	7,94	Forest Zone	neutral	Lo
CI9	Gontougo	-2,97	8,22	Savannah Zone	acidic	SaLo

Supplementary Table S2. Mean of environmental and physico-chemical characteristics of vegetation types soils. Climatic data are from the CHELSA database, from 1987 to 2013. Prec=precipitation, Temp=temperature. Statistical comparisons using Kruskal-Wallis test, followed by a Wilcoxon rank sum test (with a Benjamini-Hochberg correction) were done among the three types of vegetation. P-values are in bold and statistical significances are shown by '*', '**', and '***' for p<0.05, p<0.01, and p<0.0001, respectively. Letters in bold show the result of the Wilcoxon test for groups that are statistically different. Comparisons were done by line

	Forest Zone	Forest-Savannah contact zone	Savannah Zone	p-value
Chemical elements				
pH	6 ± 0.71	6.3 ± 0.36	6.1 ± 0.34	0.24
C (%)	1.4 ± 0.46 **a**	1.2 ± 0.36 **ab**	0.98 ± 0.42 **b**	**0.01***
N (%)	0.16 ± 0.06 **a**	0.11 ± 0.03 **ab**	0.079 ± 0.04 **b**	**2.59e-04*****
K (mg/kg)	74 ± 62	77 ± 58	49 ± 21	0.76
Ca (mg/kg)	1e+03 ± 8.7e+02	7.9e+02 ± 4.3e+02	7e+02 ± 3.6e+02	0.7
Mg (mg/kg)	1.8e+02 ± 1.3e+02	1.5e+02 ± 53	1.4e+02 ± 63	0.76
Na (mg/kg)	26 ± 18	22 ± 1.8	20 ± 3.3	0.63
P (mg/kg)	19 ± 29	11 ± 6.9	12 ± 5.6	0.51
Mn (mg/kg)	62 ± 79	52 ± 25	70 ± 40	0.18
Fe (mg/kg)	75 ± 40	63 ± 12	65 ± 33	0.55
Al (mg/kg)	3.6e+02 ± 1.7e+02	4.7e+02 ± 1e+02	4.6e+02 ± 2e+02	0.09
Granulometry				
Sand (%)	64 ± 14	71 ± 3.6	74 ± 13	0.55
Silt (%)	15 ± 11	9.1 ± 3.6	11 ± 9.2	0.5
Clay (%)	21 ± 7.1	20 ± 5.4	16 ± 6.4	0.05
Climate data				
Prec (mm)	1.4e+03 ± 2.5e+02 **a**	1.2e+03 ± 50 **ab**	1.2e+03 ± 1.2e+02 **b**	**3.51e-03****
Temp (°C)	26 ± 0.56 **a**	26 ± 0.19 **b**	26 ± 0.53 **b**	**1.62e-03****

Supplementary Table S3. Quality metrics of the V4-V5 16S rDNA sequencing data.

**a. Total number of sequences before and after bioinformatics processing and quality filtering**	
Total number of raw reads before QC	3.645.012
Total number of clean reads after QC	3.175.778

**b. Size distribution of clean reads (bp)**	Sequences	Percentage	ASV	Percentage
0-280	25	<0.01%	17	<1%
280-300	398	0.01%	130	<1%
300-320	183	<0.01%	130	<1%
320-340	267	<0.01%	173	<1%
340-360	381	0.01%	191	<1%
360-380	3137425	98.79%	22091	88.96%
380-400	34877	1.10%	1018	4.10%
400-420	883	0.03%	263	1.06%
420-440	300	<0.01%	199	<1%
440-460	353	0.01%	186	<1%
460-480	215	<0.01%	126	<1%
480-500	199	<0.01%	141	<1%
500-540	272	<0.01%	167	<1%

Total	*3175778*	*100%*	*24832*	*100%*

a: Total number of reads from the 45 samples before and after quality control (QC)
b: Lengths of high quality sequences and ASVs obtained after taxonomic classification splitted by size ranges

Supplementary Table S4. Wilcoxon rank sum test comparisons among phyla. Std=standard deviation. Min=mimimum value of value. Max=maximum value of abundance. Significant differences are shown by letters denoting the corresponding group of significance.

	Phyla	Relative Abundance	Relative Occurrence	Means	Std	Min	Max	Significant differences
1	Proteobacteria	31.64	100	21296.02	5246.80	11514	30502	a
2	Acidobacteria	17.19	100	11571.04	3120.87	7046	22562	b
3	Actinobacteria	17.14	100	11537.96	5045.34	5270	27047	b
4	Bacteroidetes	8.90	100	5993.09	1682.16	2098	10141	c
5	Verrucomicrobia	6.06	100	4081.09	2096.38	721	8657	d
6	Planctomycetes	5.17	100	3480.13	1205.32	1578	7915	d
7	Chloroflexi	5.07	100	3412.33	1884.25	1192	11812	d
8	Firmicutes	3.36	100	2263.31	1393.80	588	7608	e
9	candidate division WPS-1	2.35	100	1580.80	785.02	653	3517	ef
10	Gemmatimonadetes	1.44	100	967.44	404.62	310	1845	fg
11	Armatimonadetes	0.44	100	293.82	167.36	70	714	g
12	Nitrospirae	0.30	100	199.64	167.85	36	714	g
13	Latescibacteria	0.25	91.11	169.11	121.21	0	514	g
14	Cyanobacteria	0.2	97.78	166	102	0	424	g
15	Thermodesulfobacteria	0.15	97.78	99.82	99.23	0	629	g
16	candidate division WPS-2	0.10	73.33	65.93	89.89	0	448	g
17	Elusimicrobia	0.06	60.00	39.24	41.50	0	143	g
18	Chlamydiae	0.06	57.78	37.11	52.37	0	211	g
19	Rhodothermaeota	0.05	60.00	31.64	42.03	0	150	g
20	Thermotogae	0.04	22.22	25.58	147.04	0	987	g
21	Acetothermia	0.03	46.67	18.91	28.96	0	111	g
22	Aminicenantes	0.03	82.22	18.13	15.73	0	62	g
23	Aquificae	0.03	66.67	17.60	19.75	0	77	g
24	Ignavibacteriae	0.03	75.56	17.33	16.16	0	63	g
25	Deinococcus-Thermus	0.02	28.89	14.89	29.69	0	123	g
26	Synergistetes	0.02	55.56	13.40	22.30	0	107	g
27	Deferribacteres	0.02	42.22	13.13	19.90	0	85	g
28	candidate division ZB3	0.02	40.00	10.58	16.72	0	62	g
29	Parcubacteria	0.01	28.89	9.04	24.62	0	143	g
30	Omnitrophica	0.01	60.00	7.27	9.35	0	47	g
31	Abditibacteriota	0.01	35.56	5.89	17.15	0	111	g
32	Campilobacterota	0.01	33.33	4.58	11.74	0	59	g
33	Candidatus Saccharibacteria	0.00	15.56	2.93	8.25	0	38	g
34	BRC1	0.00	24.44	2.76	5.12	0	18	g
35	Kiritimatiellaeota	0.00	13.33	2.22	7.60	0	37	g
36	Microgenomates	0.00	37.78	1.80	3.36	0	16	g
37	Hydrogenedentes	0.00	13.33	1.58	5.17	0	29	g
38	Fibrobacteres	0.00	6.67	1.31	5.10	0	25	g
39	Tenericutes	0.00	31.11	1.31	2.93	0	14	g
40	Spirochaetes	0.00	24.44	0.71	2.04	0	12	g
41	Balneolaeota	0.00	11.11	0.42	1.75	0	11	g
42	Candidatus Calescamantes	0.00	4.44	0.13	0.66	0	4	g
43	Fusobacteria	0.00	4.44	0.13	0.63	0	3	g
44	Marinimicrobia	0.00	2.22	0.11	0.75	0	5	g
45	Lentisphaerae	0.00	2.22	0.09	0.60	0	4	g
46	Cloacimonetes	0.00	2.22	0.04	0.30	0	2	g
47	SR1	0.00	2.22	0.04	0.30	0	2	g

48	Chrysiogenetes	0.00	2.22	0.02	0.15	0	1	g

Supplementary Table S5. Major genera identifed in 100% of the soils with a proportion of sequences greater than 0.5%.

Phyla	Classes	Order	Family	Genus	Relative abundance
Acidobacteria	Acidobacteria_Gp1	Unknown	Unknown	*Gp1*	1.84
	Acidobacteria_Gp16	Unknown	Unknown	*Gp16*	0.51
	Acidobacteria_Gp2	Unknown	Unknown	*Gp2*	1.01
	Acidobacteria_Gp3	Unknown	Unknown	*Gp3*	1.25
	Acidobacteria_Gp4	Unknown	Unknown	*Gp4*	2.35
	Acidobacteria_Gp6	Unknown	Unknown	*Gp6*	5.64
	Vicinamibacteria	Vicinamibacterales	Vicinamibacteraceae	*Luteitalea*	0.58
Actinobacteria	Acidimicrobiia	Acidimicrobiales	Acidimicrobiales_ incertae_ sedis	*Aciditerrimonas*	0.63
	Actinobacteria	Mycobacteriales	Mycobacteriaceae	*Mycobacterium*	0.53
		Propionibacteriales	Nocardioidaceae	*Nocardioides*	0.58
		Streptosporangiales	Thermomonosporaceae	*Actinoallomurus*	0.73
	Thermoleophilia	Gaiellales	Gaiellaceae	*Gaiella*	4.41
		Solirubrobacterales	Baekduiaceae	*Baekduia*	1.27
			Conexibacteraceae	*Conexibacter*	0.77
			Parviterribacteraceae	*Parviterribacter*	0.57
			Solirubrobacteraceae	*Solirubrobacter*	0.60
Bacteroidetes	Chitinophagia	Chitinophagales	Chitinophagaceae	*Flavisolibacter*	1.21
				Flavitalea	0.50
				Terrimonas	1.74
	Flavobacteriia	Flavobacteriales	Flavobacteriaceae	*Flavobacterium*	0.55
candidate division WPS-1	Unknown	Unknown	Unknown	*WPS-1_genera_ incertae_sedis*	2.35
Chloroflexi	Chloroflexia	Chloroflexales	Roseiflexaceae	*Roseiflexus*	1.18
	Ktedonobacteria	Ktedonobacterales	Ktedonobacteraceae	*Ktedonobacter*	0.76
Firmicutes	Bacilli	Bacillales	Bacillaceae 1	*Bacillus*	0.66
				Neobacillus	0.59
Gemmatimonadetes	Gemmatimonadetes	Gemmatimonadales	Gemmatimonadaceae	*Roseisolibacter*	0.81
Planctomycetes	Planctomycetacia	Planctomycetales	Gemmataceae	*Fimbriiglobus*	1.83
Proteobacteria	Alphaproteobacteria	Rhizobiales	Blastochloridaceae	*Blastochloris*	0.73
			Bradyrhizobiaceae	*Bradyrhizobium*	1.96
				Pseudolabrys	1.01
				Pseudorhodoplanes	2.09
				Rhodoplanes	2.24
			Methylobacteriaceae	*Microvirga*	0.70
		Rhodospirillales	Rhodospirillaceae	*Aliidongia*	0.60
		Sphingomonadales	Sphingomonadaceae	*Sphingomonas*	0.70
	Betaproteobacteria	Burkholderiales	Comamonadaceae	*Tepidimonas*	0.74
		Nitrosomonadales	Methylophilaceae	*Novimethylophilus*	0.66
			Sterolibacteriaceae	*Sulfuritalea*	0.51
	Deltaproteobacteria	Myxococcales	Haliangiaceae	*Haliangium*	0.64
	Gammaproteobacteria	Gammaproteobacteria_incertae_ sedis	Unknown	*Acidibacter*	1.16
		Nevskiales	Steroidobacteraceae	*Povalibacter*	0.53
Verrucomicrobia	Spartobacteria	Unknown	Unknown	*Spartobacteria_genera _incertae_sedis*	3.86
	Subdivision3	Unknown	Unknown	*Subdivision3_genera _incertae_sedis*	1.80

Supplementary Table S6. Kruskal-Wallis multiple comparison test on the abundance of the core bacteriobiome by vegetation type. Statistical comparisons using the Wilcoxon test was done among the three types of vegetation. P-values are in bold and statistical significances are shown by '*', '**', and '***' for p<0.05, p<0.01, and p<0.0001, respectively. Letters in bold show the result of the Wilcoxon test for groups that are statistically different. Comparisons were done by line.

	Forest Zone	Forest-Savannah contact zone	Savannah Zone	p-values
Bradyrhizobium	1.4e+03 ± 8e+02	1.2e+03 ± 6.8e+02	1.3e+03 ± 4.6e+02	0.94
Gaiella	2.8e+03 ± 1.6e+03	4.1e+03 ± 2.7e+03	2.8e+03 ± 1.7e+03	0.61
Gp3	8.2e+02 ± 3.9e+02	7.4e+02 ± 2.7e+02	9e+02 ± 4e+02	0.66
Gp4	1.1e+03 ± 6.7e+02 **a**	1.8e+03 ± 1.1e+03 **ab**	2.1e+03 ± 8.9e+02 **b**	**1.5e-03***
Gp6	4.2e+03 ± 1.8e+03	4e+03 ± 1.4e+03	3.2e+03 ± 1.2e+03	0.24
Pseudorhodoplanes	1.6e+03 ± 8.7e+02	1.6e+03 ± 7.7e+02	1.1e+03 ± 4.1e+02	0.15
Rhodoplanes	1.6e+03 ± 5.1e+02	1.8e+03 ± 7.1e+02	1.4e+03 ± 4.9e+02	0.3
Spartobacteria_genera_incertae_sedis	2.5e+03 ± 1.6e+03	4e+03 ± 1.9e+03	2.3e+03 ± 1.3e+03	0.11
Subdivision3_genera_incertae_sedis	1.4e+03 ± 9.8e+02	8.3e+02 ± 2.1e+02	1e+03 ± 4.9e+02	0.28
WPS-1_genera_incertae_sedis	1.1e+03 ± 3.6e+02 **a**	1.6e+03 ± 8.1e+02 **ab**	2.3e+03 ± 7.2e+02 **b**	**2.4e-05*****

Supplementary Table S7. Kruskal-Wallis multiple comparison test on the abundance of the core bacteriobiome by soil groups. Statistical comparisons using the Wilcoxon test was done among the three groups of soils. P-values are in bold and statistical significances are shown by '*', '**', and '***' for p<0.05, p<0.01, and p<0.0001, respectively. Letters in bold show the result of the Wilcoxon test for groups that are statistically different. Comparisons were done by line.

	Gr1	Gr2	Gr3	p-values
Bradyrhizobium	1.3e+03 ± 6.7e+02 **a**	1.9e+03 ± 5.7e+02 **b**	1.1e+03 ± 5.4e+02 **a**	**1.9e-03***
Gaiella	6.1e+03 ± 1.2e+03 **a**	1.7e+03 ± 6.6e+02 **b**	2.6e+03 ± 9.6e+02 **c**	**3.8e-06*****
Gp3	6.6e+02 ± 2.5e+02 **a**	1.2e+03 ± 3.4e+02 **b**	7e+02 ± 2.9e+02 **a**	**3.1e-04*****
Gp4	1.7e+03 ± 1.2e+03 **ab**	9.3e+02 ± 4.4e+02 **a**	1.9e+03 ± 8.7e+02 **b**	**9.1e-03***
Gp6	4.2e+03 ± 1.4e+03 **a**	2.7e+03 ± 1.3e+03 **b**	4.2e+03 ± 1.7e+03 **a**	**0.01***
Pseudorhodoplanes	2.3e+03 ± 1.1e+03 **a**	1.5e+03 ± 4.3e+02 **a**	1.1e+03 ± 4.4e+02 **b**	**1.3e-03***
Rhodoplanes	2.2e+03 ± 4.6e+02 **a**	1.4e+03 ± 4.3e+02 **b**	1.3e+03 ± 3.9e+02 **b**	**7.1e-04*****
Spartobacteria_genera_incertae_sedis	2.5e+03 ± 8.6e+02 **ab**	4e+03 ± 1.9e+03 **a**	1.9e+03 ± 1.1e+03 **b**	**4e-03***
Subdivision3_genera_incertae_sedis	7.7e+02 ± 2e+02 **a**	1.9e+03 ± 1.1e+03 **b**	1e+03 ± 4.5e+02 **a**	**5.2e-03***
WPS-1_genera_incertae_sedis	1.7e+03 ± 8.4e+02 **ab**	1.1e+03 ± 6.4e+02 **a**	1.8e+03 ± 7.8e+02 **b**	**0.02***

Supplementary Table S8. Spearman's correlation coefficients between core genera isolated from Côte d'Ivoire soils. Values in bold represent strong correlations, here defined when the correlation coefficient is >0.5 or <-0.5. Statistical significances are shown by '*', '**', and '***' for p<0.05, p<0.01, and p<0.0001, respectively.

	Gaiella	*Brady rhizobium*	*Gp3*	*Gp4*	*Gp6*	*Pseudo Rhodo planes*	*Rhodo planes*	*Sparto bacteria_ genera_ incertae_sedis*	*Subdivision3_ genera_ incertae_ sedis*
Bradyrhizobium	-0.15								
Gp3	-0.35*	**0.68*** **							
Gp4	0.19	-0.37*	-0.11						
Gp6	0.33*	**-0.58*** **	**-0.69*** **	0.34*					
Pseudorhodoplanes	0.34*	**0.50*** **	0.20	-0.28	-0.01				
Rhodoplanes	**0.55*** **	0.39**	0.01	-0.12	0.12	**0.76*** **			
Spartobacteria_ genera_incertae_ sedis	-0.07	**0.67*** **	0.39**	-0.26	-0.25	**0.51*** **	0.44**		
Subdivision3_genera _incertae_sedis	-0.40**	**0.60*** **	0.45**	-0.21	-0.15	0.30*	0.05	**0.55*** **	
WPS.1_genera_ incertae_sedis	0.21	-0.19	-0.14	**0.71*** **	0.18	-0.18	-0.12	-0.01	-0.11

Supplementary Table S9. Summary of the relative energy metabolism function for each genus and the module relative abundance in each energy metabolism pathway level for each genus.

Genera	Energy Metabolism (Level 3)	Function relative abundance (%)	Modules in Energy Metabolism function	Module relative abundance (%)
Bradyrhizobium	Carbon fixation	52.45	3-Hydroxypropionate bi-cycle	38.29
			CAM (Crassulacean acid metabolism), dark	5.52
			CAM (Crassulacean acid metabolism), light	6.87
			Reductive pentose phosphate cycle (Calvin cycle) / Reductive pentose phosphate cycle, ribulose-5P => glyceraldehyde-3P	15.00
			Reductive pentose phosphate cycle (Calvin cycle) / Reductive pentose phosphate cycle, glyceraldehyde-3P => ribulose-5P	29.25
			Phosphate acetyltransferase-acetate kinase pathway, acetyl-CoA	5.07
	Nitrogen metabolism	11.51	Assimilatory nitrate reduction, nitrate => ammonia	5.55
			Dissimilatory nitrate reduction, nitrate => ammonia	19.60
			Denitrification, nitrate => nitrogen	16.04
			Dissimilatory nitrate reduction, nitrate => ammonia / Denitrification, nitrate => nitrogen	23.29
			Nitrogen fixation, nitrogen => ammonia	35.53
	Sulfur metabolism	29.43	Assimilatory sulfate reduction, sulfate => H2S	37.76
			Thiosulfate oxidation by SOX complex, thiosulfate => sulfate	62.24
	Symbiosis	6.61	Nodulation	100.00
Gaiella	Carbon fixation	88.80	3-Hydroxypropionate bi-cycle	48.41
			CAM (Crassulacean acid metabolism), dark	5.77
			CAM (Crassulacean acid metabolism), light	5.67
			Reductive pentose phosphate cycle (Calvin cycle) / Reductive pentose phosphate cycle, ribulose-5P => glyceraldehyde-3P	11.45
			Reductive pentose phosphate cycle (Calvin cycle) / Reductive pentose phosphate cycle, glyceraldehyde-3P => ribulose-5P	28.42
			Phosphate acetyltransferase-acetate kinase pathway, acetyl-CoA	0.27
	Nitrogen metabolism	0.32	Assimilatory nitrate reduction, nitrate => ammonia	8.74
			Dissimilatory nitrate reduction, nitrate => ammonia	25.60
			Denitrification, nitrate => nitrogen	6.91
			Dissimilatory nitrate reduction, nitrate => ammonia / Denitrification, nitrate => nitrogen	25.62
			Nitrogen fixation, nitrogen => ammonia	33.14
	Sulfur metabolism	10.88	Assimilatory sulfate reduction, sulfate => H2S	99.94
			Dissimilatory sulfate reduction, sulfate => H2S	0.00
			Dissimilatory sulfate reduction, sulfate => H2S / Assimilatory sulfate reduction, sulfate => H2S	0.06
Gp3	Carbon fixation	81.75	3-Hydroxypropionate bi-cycle	47.35
			CAM (Crassulacean acid metabolism), dark	4.58
			CAM (Crassulacean acid metabolism), light	5.59
			Reductive pentose phosphate cycle (Calvin cycle) / Reductive pentose phosphate cycle, ribulose-5P => glyceraldehyde-3P	11.18
			Reductive pentose phosphate cycle (Calvin cycle) / Reductive pentose phosphate cycle, glyceraldehyde-3P => ribulose-5P	24.70
			Phosphate acetyltransferase-acetate kinase pathway, acetyl-CoA	6.60
	Nitrogen metabolism	1.89	Dissimilatory nitrate reduction, nitrate => ammonia	88.84
			Nitrogen fixation, nitrogen => ammonia	11.16

	Sulfur metabolism	16.36	Assimilatory sulfate reduction, sulfate => H2S	100.00
Gp4	Carbon fixation	92.01	3-Hydroxypropionate bi-cycle	53.45
			CAM (Crassulacean acid metabolism), dark	7.86
			CAM (Crassulacean acid metabolism), light	4.42
			Reductive pentose phosphate cycle (Calvin cycle) / Reductive pentose phosphate cycle, ribulose-5P => glyceraldehyde-3P	8.83
			Reductive pentose phosphate cycle (Calvin cycle) / Reductive pentose phosphate cycle, glyceraldehyde-3P => ribulose-5P	17.29
			Phosphate acetyltransferase-acetate kinase pathway, acetyl-CoA	8.15
	Nitrogen metabolism	6.72	Dissimilatory nitrate reduction, nitrate => ammonia	51.26
			Dissimilatory nitrate reduction, nitrate => ammonia / Denitrification, nitrate => nitrogen	48.74
	Sulfur metabolism	1.27	Assimilatory sulfate reduction, sulfate => H2S	100.00
Gp6	Carbon fixation	95.71	3-Hydroxypropionate bi-cycle	58.39
			CAM (Crassulacean acid metabolism), dark	4.39
			CAM (Crassulacean acid metabolism), light	4.65
			Reductive pentose phosphate cycle (Calvin cycle) / Reductive pentose phosphate cycle, ribulose-5P => glyceraldehyde-3P	9.30
			Reductive pentose phosphate cycle (Calvin cycle) / Reductive pentose phosphate cycle, glyceraldehyde-3P => ribulose-5P	18.60
			Phosphate acetyltransferase-acetate kinase pathway, acetyl-CoA	4.67
	Nitrogen metabolism	4.17	Dissimilatory nitrate reduction, nitrate => ammonia	99.98
			Denitrification, nitrate => nitrogen	0.00
			Dissimilatory nitrate reduction, nitrate => ammonia / Denitrification, nitrate => nitrogen	0.01
			Nitrogen fixation, nitrogen => ammonia	0.01
	Sulfur metabolism	0.12	Assimilatory sulfate reduction, sulfate => H2S	100.00
Pseudorhodoplanes	Carbon fixation	68.44	3-Hydroxypropionate bi-cycle	43.09
			CAM (Crassulacean acid metabolism), dark	6.87
			CAM (Crassulacean acid metabolism), light	8.59
			Reductive pentose phosphate cycle (Calvin cycle) / Reductive pentose phosphate cycle, ribulose-5P => glyceraldehyde-3P	15.61
			Reductive pentose phosphate cycle (Calvin cycle) / Reductive pentose phosphate cycle, glyceraldehyde-3P => ribulose-5P	20.66
			Phosphate acetyltransferase-acetate kinase pathway, acetyl-CoA	5.18
	Nitrogen metabolism	7.04	Assimilatory nitrate reduction, nitrate => ammonia	33.33
			Dissimilatory nitrate reduction, nitrate => ammonia	33.33
			Dissimilatory nitrate reduction, nitrate => ammonia / Denitrification, nitrate => nitrogen	33.33
	Sulfur metabolism	24.53	Assimilatory sulfate reduction, sulfate => H2S	47.99
			Thiosulfate oxidation by SOX complex, thiosulfate => sulfate	52.01
Rhodoplanes	Carbon fixation	71.09	3-Hydroxypropionate bi-cycle	41.24
			CAM (Crassulacean acid metabolism), dark	6.37
			CAM (Crassulacean acid metabolism), light	8.47
			Reductive pentose phosphate cycle (Calvin cycle) / Reductive pentose phosphate cycle, ribulose-5P => glyceraldehyde-3P	17.91
			Reductive pentose phosphate cycle (Calvin cycle) / Reductive pentose phosphate cycle, glyceraldehyde-3P => ribulose-5P	20.44
			Phosphate acetyltransferase-acetate kinase pathway, acetyl-CoA	5.56
	Nitrogen metabolism	6.76	Assimilatory nitrate reduction, nitrate => ammonia	33.33
			Dissimilatory nitrate reduction, nitrate => ammonia	33.33

			Dissimilatory nitrate reduction, nitrate => ammonia / Denitrification, nitrate => nitrogen	33.33
			Nitrogen fixation, nitrogen => ammonia	0.00
	Sulfur metabolism	22.15	Assimilatory sulfate reduction, sulfate => H2S	51.29
			Thiosulfate oxidation by SOX complex, thiosulfate => sulfate	48.71
Spartobacteria_ genera_incertae_ sedis	Carbon fixation	78.26	3-Hydroxypropionate bi-cycle	44.44
			CAM (Crassulacean acid metabolism), dark	11.11
			CAM (Crassulacean acid metabolism), light	5.56
			Reductive pentose phosphate cycle (Calvin cycle) / Reductive pentose phosphate cycle, ribulose-5P => glyceraldehyde-3P	11.11
			Reductive pentose phosphate cycle (Calvin cycle) / Reductive pentose phosphate cycle, glyceraldehyde-3P => ribulose-5P	16.67
			Phosphate acetyltransferase-acetate kinase pathway, acetyl-CoA	11.11
	Sulfur metabolism	21.74	Assimilatory sulfate reduction, sulfate => H2S	100.00
Subdivision3_ genera_incertae_ sedis	Carbon fixation	78.35	3-Hydroxypropionate bi-cycle	44.46
			CAM (Crassulacean acid metabolism), dark	11.08
			CAM (Crassulacean acid metabolism), light	5.56
			Reductive pentose phosphate cycle (Calvin cycle) / Reductive pentose phosphate cycle, ribulose-5P => glyceraldehyde-3P	11.13
			Reductive pentose phosphate cycle (Calvin cycle) / Reductive pentose phosphate cycle, glyceraldehyde-3P => ribulose-5P	16.69
			Phosphate acetyltransferase-acetate kinase pathway, acetyl-CoA	11.08
	Sulfur metabolism	21.65	Assimilatory sulfate reduction, sulfate => H2S	100.00
WPS-1_genera_ incertae_sedis	Carbon fixation	91.98	3-Hydroxypropionate bi-cycle	38.61
			CAM (Crassulacean acid metabolism), dark	6.32
			CAM (Crassulacean acid metabolism), light	6.51
			Reductive pentose phosphate cycle (Calvin cycle) / Reductive pentose phosphate cycle, ribulose-5P => glyceraldehyde-3P	12.64
			Reductive pentose phosphate cycle (Calvin cycle) / Reductive pentose phosphate cycle, glyceraldehyde-3P => ribulose-5P	27.22
			Phosphate acetyltransferase-acetate kinase pathway, acetyl-CoA	8.71
	Nitrogen metabolism	0.16	Assimilatory nitrate reduction, nitrate => ammonia	24.97
			Dissimilatory nitrate reduction, nitrate => ammonia	50.04
			Denitrification, nitrate => nitrogen	0.03
			Dissimilatory nitrate reduction, nitrate => ammonia / Denitrification, nitrate => nitrogen	24.97
	Sulfur metabolism	7.85	Assimilatory sulfate reduction, sulfate => H2S	100.00

Supplementary Table S10. Mean of environmental and physico-chemical characteristics of the groups of soils. Climatic data are from the CHELSA database, from 1987 to 2013. Prec=precipitation, Temp=temperature. Statistical comparisons using Kruskal-Wallis test, followed by a Wilcoxon rank sum test (with a Benjamini-Hochberg correction) were done among the three groups of soils. P-values are in bold and statistical significances are shown by ‘*’, ‘**’, and ‘***’ for p<0.05, p<0.01, and p<0.0001, respectively. Letters in bold show the result of the Wilcoxon test for groups that are statistically different. Comparisons were done by line.

	Gr1	Gr2	Gr3	p-value
Chemical elements				
pH	6.1 ± 0.32 **a**	5.5 ± 0.33 **b**	6.3 ± 0.54 **a**	**1.78e-04*** **
C (%)	1.2 ± 0.35	1.2 ± 0.46	1.3 ± 0.53	0.96
N (%)	0.12 ± 0.05	0.13 ± 0.06	0.13 ± 0.08	0.93
K (mg/kg)	58 ± 26 **a**	34 ± 18 **b**	82 ± 59 **a**	**0.01***
Ca (mg/kg)	8.4e+02 ± 4.1e+02 **a**	4.5e+02 ± 2.8e+02 **b**	1.1e+03 ± 7.9e+02 **a**	**4.7e-03****
Mg (mg/kg)	1.5e+02 ± 58	1.1e+02 ± 64	1.9e+02 ± 1.2e+02	0.06
Na (mg/kg)	20 ± 2.5	20 ± 4.4	26 ± 18	0.38
P (mg/kg)	11 ± 8.6 **a**	11 ± 18 **ab**	19 ± 25 **a**	**0.02***
Mn (mg/kg)	53 ± 32 **a**	20 ± 24 **b**	89 ± 69 **a**	**1.1e-04*** **
Fe (mg/kg)	58 ± 14 **a**	95 ± 48 **b**	62 ± 27 **a**	**0.02***
Al (mg/kg)	5.1e+02 ± 2.2e+02	4.1e+02 ± 1.8e+02	3.8e+02 ± 1.6e+02	0.36
Granulometry				
Sand (%)	70 ± 6.9	68 ± 12	69 ± 16	0.8
Silt (%)	8.6 ± 3.3	14 ± 10	13 ± 11	0.69
Clay (%)	21 ± 6.1	18 ± 6	18 ± 7.7	0.35
Climate data				
Prec (mm)	1.3e+03 ± 81 **a**	1.4e+03 ± 3.2e+02 **ab**	1.2e+03 ± 1.3e+02 **b**	**8.02e-03****
Temp (°C)	26 ± 0.52 **a**	26 ± 0.6 **a**	26 ± 0.45 **b**	**1.34e-03****
